# New GMP manufacturing processes to obtain thermostable HIV-1 gp41 virosomes under solid forms for various mucosal vaccination routes

**DOI:** 10.1038/s41541-020-0190-9

**Published:** 2020-05-18

**Authors:** Mario Amacker, Charli Smardon, Laura Mason, Jack Sorrell, Kirk Jeffery, Michael Adler, Farien Bhoelan, Olga Belova, Mark Spengler, Beena Punnamoottil, Markus Schwaller, Olivia Bonduelle, Behazine Combadière, Toon Stegmann, Andrew Naylor, Richard Johnson, Desmond Wong, Sylvain Fleury

**Affiliations:** 1 Mymetics SA, 4 Route de la Corniche, 1066 Epalinges, Switzerland; 2 Catalent U.K. Swindon Zydis Limited, Frankland Road, Blagrove, Swindon, SN5 8RU Wiltshire UK; 3 Upperton Limited, Albert Einstein Centre, Nottingham Science Park, Nottingham, NG7 2TN UK; 4 Chimera Biotec GmbH, Emil-Figge-Strasse 76A, 44227 Dortmund, Germany; 5 Mymetics BV, JH Oortweg 21, 2333 CH Leiden, The Netherlands; 6 Bachem AG, Hauptstrasse 144, 4416 Bubendorf, Switzerland; 7 Centre d’immunologie et des Maladies Infectieuses, Sorbonne Université, INSERM U1135, Paris, France

**Keywords:** Biotechnology, Immunology, Infectious diseases

## Abstract

The main objective of the MACIVIVA European consortium was to develop new Good Manufacturing Practice pilot lines for manufacturing thermostable vaccines with stabilized antigens on influenza virosomes as enveloped virus-like particles. The HIV-1 gp41-derived antigens anchored in the virosome membrane, along with the adjuvant 3M-052 (TLR7/8 agonist) on the same particle, served as a candidate vaccine for the proof of concept for establishing manufacturing processes, which can be directly applied or adapted to other virosomal vaccines or lipid-based particles. Heat spray-dried powders suitable for nasal or oral delivery, and freeze-dried sublingual tablets were successfully developed as solid dosage forms for mucosal vaccination. The antigenic properties of vaccinal antigens with key gp41 epitopes were maintained, preserving the original immunogenicity of the starting liquid form, and also when solid forms were exposed to high temperature (40 °C) for up to 3 months, with minimal antigen and adjuvant content variation. Virosomes reconstituted from the powder forms remained as free particles with similar size, virosome uptake by antigen-presenting cells in vitro was comparable to virosomes from the liquid form, and the presence of excipients specific to each solid form did not prevent virosome transport to the draining lymph nodes of immunized mice. Virosome integrity was also preserved during exposure to <−15 °C, mimicking accidental freezing conditions. These “ready to use and all-in-one” thermostable needle-free virosomal HIV-1 mucosal vaccines offer the advantage of simplified logistics with a lower dependence on the cold chain during shipments and distribution.

## Introduction

The majority of the world population lives in warm regions, and for low and middle-income countries, maintaining the cold chain for preserving biological products is challenging due to unreliable electricity access and inadequate or limited storage facilities. Developing stabilized liquid or solid vaccine dosage forms for improving their thermostability is part of the solution for these countries but it is a daunting task. Low moisture content has been already identified as a promising approach for stabilizing vaccines and virosome-based vaccines^1–8^ but most of these new powder form vaccines are still vulnerable to high temperatures.

Virosomes are a type of subunit vaccine displaying lipid-anchored antigens at the surface of lipid-based particles with an empty lumen, acting as efficient antigen delivery vehicles^9–11^ with a mean diameter generally ranging from 80 to 120 nm. Because they have a similar size and shape to viruses, they belong to the enveloped virus-like particles (eVLP) family. Virosomes are synthetic particles that are in vitro assembled in a cell-free system, with part of their lipid membrane originating from purified viral membrane components (from the influenza virus for this vaccine candidate). In common with other VLPs, they lack nucleic acid and are non-infectious, as for other VLPs. Antigens are free to move in *cis* and/or rotate on its axis at the virosome surface, leading to variable distance between antigens that may contribute to expose most if not all potential epitopes in an optimal way. These properties are key differentiators with the more standard non-enveloped VLPs forming a protein core, with vaccinal antigens that have fixed positions in the VLP structure with very limited movement, which may potentially reduce the access to certain regions, particularly if antigens are very close to each other.

Liquid virosomes are sensitive to heat and freezing, causing irreversible damage to the particles and/or antigens that destroys bioactivity of the vaccine. Therefore, permanent cooling of virosomal vaccines, as for many liquid vaccines, is still a fundamental requisite for preserving their bioactivity. The consortium MACIVIVA is the acronym for “**M**anufacturing process for **C**old chain **I**ndependent **Vi**rosome-based **Va**ccines”. The group used the promising human immunodeficiency virus type 1 (HIV-1) candidate vaccine MYM-V202 based on gp41-derived antigens anchored on virosomes as a lipid-based test product under a liquid form and proof of concept for establishing new Good Manufacturing Practice (GMP) pilot lines for obtaining thermostable mucosal solid vaccine forms by spray drying or lyophilization.

The HIV-1 is mainly transmitted through sexual contact^12^ with the genital and gastrointestinal tracts as the main entry points. An effective HIV-1 vaccine must be capable of eliciting mucosal innate and adaptive immunity in these different entry doors for an efficient front-line defense against HIV-1^13–15^. With the existence of a common mucosal system implicating the respiratory, genital, and gastrointestinal mucosa, innate cells such as NK cells and antigen-specific T and B lymphocytes induced at a given mucosal site can also migrate and seed other distant mucosal tissues through the mucosal network for promoting a generalized mucosal immune response^16^. This is why vaccine strategies with immunization regimens involving mucosal administration routes^17–19^ are expected to be more efficient to induce higher numbers of mucosal resident immune cells in distinct mucosal tissues that can rapidly expand for fighting the local infection or the arrival of new mucosal pathogens responsible for early acquisition and infection events.

This contrasts with the traditional parenteral immunization involving the intramuscular (IM) and subcutaneous (SC) routes that generally elicits circulating B and T cells that remain mostly in the periphery, with generally fewer numbers reaching the mucosal tissues, and consequently a lower number of mucosal resident antigen-specific immune cells as front-line defense. This offers, as a consequence, a short-time window infection opportunity for certain invading mucosal pathogens like the HIV-1, which rapidly replicates within 24–48 h in target cells present in the mucosal tissues, without being concerned by the weak vaccine-induced patrolling immune defense against HIV-1 at the mucosa. These mucosal pathogens then spread either to other target cells present at the mucosal level and/or migrate to the lymph nodes or reach the blood circulation prior to the reinforcement arrival from the adaptive immune system coming from the periphery. Preventing this very early mucosal infection is particularly crucial for pathogens capable of creating active or latent cell reservoirs in its host that are often invisible to the host immune system and can be a discontinuous or continuous source of newly produced pathogens, as reported for HIV-1 or herpes simplex viruses^20,21^. Meanwhile, there are some exceptions with vaccines delivered by parenteral vaccination that may offer protection against certain mucosal pathogens^22–26^. Today, subunit vaccines generally involve a single mucosal administration route^27,28^ or a single parenteral route^29–31^, and more recently combined parenteral routes^32^ or sometimes a mucosal vaccine combined with an intramuscular route^22,33^. However, within the compartmentalized mucosal immune system, the induction of strong immune responses in various distant mucosal tissues is challenging.

HIV-1 employs its viral membrane surface trimeric envelope glycoprotein gp120/gp41 to bind and infect various target cells^34^. The conserved gp41 that mediates the fusion process with the target cell membrane displays the membrane proximal ectodomain region, which is a highly conserved region recognized by broadly binding neutralizing IgG antibodies (bNAbs)^35,36^ like the 2F5^37–39^, 4E10^37,40–42^, or 10E8^43,44^. Other gp41 conserved neutralizing epitopes have been reported, such as the caveoline-1 binding motif^45^ or the QARILAV^46^ sequence recognized by serum IgA from HIV-1 highly exposed persistently seronegative (HEPS) subjects. There are also other gp41 epitopes that have induced antibodies with the ability to block HIV-1 transcytosis^47–49^ and support the antibody-dependent cellular cytotoxicity activity^50,51^. Antibodies can also promote immunoglobulin-mediated mucus entrapment of virions^52,53^ or other Fc-mediated antibody effector functions^54^, and synergies among IgG and IgA toward gp41 and/or gp120 can offer better virus inhibition and protection^50,55^. The reported conserved epitopes on gp41 make this viral protein another very attractive antigen that could be included in prophylactic HIV-1 vaccines for establishing front-line defenses at the primary mucosal entry point used by HIV-1 to prevent virus transmission, local infection, and dissemination. If passive administration of HIV-neutralizing monoclonal antibodies toward various gp41- or gp120-specific epitopes were shown to protect in non-human primate and mouse models of HIV-1 infection^56^, we could think that a vaccine combining the gp41 and gp120 antigens should also induce an optimal antibody repertoire for better protection, provided that such antigens are rationally designed to focus the vaccine-induced antibody responses on relevant protective conserved epitopes.

Previously in two independent studies, the liquid unadjuvanted bivalent virosomal HIV-1 vaccine based on two gp41-derived antigens (P1 peptide: virosome-P1 and recombinant gp41: virosome-rgp41) could induce vaginal and rectal antibodies and this early formulation was shown to efficiently protect Chinese^22^ and Indian macaques during repeated low dose vaginal challenges with SHIV_SF162P3_. Safety and immunogenicity in women were also confirmed during a Phase I trial with virosome-P1^33^. However, as for other liquid subunit vaccines stored at 4 °C, protein and peptide antigens are inherently prone to chemical modifications (oxidation, deamidation) that are revealed by high-performance liquid chromatography (HPLC) analysis and not by antigen content measured by enzyme-linked immunosorbent assay (ELISA) or Western blot, and gp41-derived antigens anchored on virosomes face the same issue. Consequently, this has represented a major hurdle for obtaining a shelf-life stability of more than 2 years with limited chemical modifications of the vaccinal gp41-derived antigens.

Because HIV-1 replicates in various mucosal tissues, an HIV subunit vaccine allowing a prime/boost approach, combining two distinct mucosal sites, could more efficiently achieve a broader mucosal tissue coverage in both men and women. This explains the strong interest in developing various new galenic virosomal formulations under thermostable solid dosages for mucosal delivery, as early studies with liquid nasal^33,57^ and sublingual (SL)^26,58^ virosomes induced systemic and mucosal antibodies.

The development of galenic formulations aimed to incorporate antigen/virosome into a suitable form of mucosal vaccine with defined chemical composition that allows the release of virosomes/antigens at the site of administration for being processed by the immune system. To further improve the vaccine-induced innate and adaptive immune responses, the 3M-052 adjuvant was anchored into the virosome membrane through its lipid tail^59^. This adjuvant is known to be thermostable in a liposomal formulation^60^. It binds to the toll-like receptor (TLR) 7/8 present in the endosomes and it is functional in an immature immune system, as found in infants and young children, as well as in a mature immune system of adults^61–63^.

The new adjuvanted HIV-1 vaccine called MYM-V202 contains two types of virosomes, one that displays P1 and the other one with rgp41, with the adjuvant anchored on the same virosome particle to minimize non-specific immune activation and further improves the vaccine tolerance and safety. With this new galenic formulation, we have also verified that the new excipients were not detrimental to virosome particles, particularly once delivered in vivo for the vaccine-induced immune responses. Experiments described in this manuscript were mainly for obtaining supportive qualitative data on the new solid vaccine forms, as the immune responses toward this HIV-1 candidate vaccine were previously characterized. The qualitative results are providing enough confidence on the new GMP manufacturing processes to confirm that the vaccine immunogenicity is preserved and the new solid vaccine forms can move into clinical development for obtaining safety and immunogenicity data after mucosal vaccination.

The acquired knowledge on virosome solid dosages may also be useful for other VLPs intended to prevent or treat other infectious or non-infectious diseases affecting mucosal tissues. The final product described in this work is a needle-free, solid dosage form vaccine “ready to use and all-in-one” contained in a single dosage for direct delivery at the mucosal site. They offer several advantages such as eliminating the reconstitution step and risk of needle injuries to improve safety, and they may improve mass vaccination and compliance due to ease of use. These thermostable vaccines would also render vaccine handling safer with simplified logistics. Those benefits should outweigh the additional cost for implementing new solid thermostable vaccine forms^64^.

## Results

### Virosome manufacturing

**Fig. 1:**
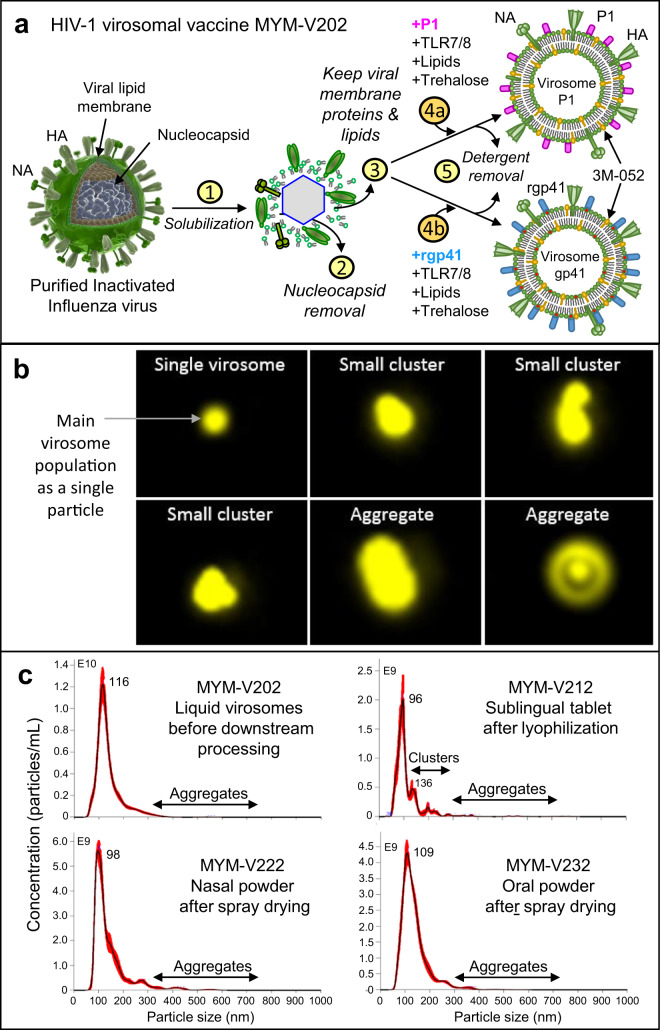
Influenza virosome-based vaccine manufacturing. **a** Production of adjuvanted virosome-P1 (MYM-V111) and virosome-rgp41 (MYM-V112): Step 1, inactivated influenza A/H1N1 are solubilized with detergent; Step 2, nucleocapsides are discarded; Step 3, the viral membrane lipids with the native influenza hemagglutinin (HA) and neuraminidase (NA) are recovered; Steps 4a and 4b, synthetic lipids with 3M-052 adjuvant and antigen P1 or rgp41 are mixed with isolated viral membrane components and trehalose; Step 5, virosomes-P1 (pink rod) and virosome-rgp41 (blue rod) are gradually assembled in vitro during the detergent removal. Each virosome is then diluted and mixed together to generate the HIV-1 liquid vaccine MYM-V202. Universal T help provided by HA/NA. **b** Amnis® ImageStream on fluorescent Dil dye-labeled virosomes (in yellow) to visualize particles. The liquid virosome population contains mostly single particles (upper left image). Reconstituted powders contain also a major population of single particles, but a minor population of few small virosome clusters and bigger aggregates are also observed. Images were enlarged in Powerpoint because original AMNIS images are only tiny dots. **c** Mean particle size and population distribution monitored by NTA for the liquid bulk vaccine (MYM-V202, upper left panel), the reconstituted sublingual tablet (MYM-V212, upper right panel), the reconstituted nasal powder (MYM-V222, lower left panel), the reconstituted oral powder (MYM-V232, lower right panel). Black arrows identify the population of small clusters 200–300 nm and aggregates >300 nm, using arbitrary cut-off.

The liquid HIV-1 candidate vaccine MYM-V202 (Fig. 1a) consists of a mixture of two distinct 3M-052 adjuvanted virosomes: the virosomes harboring P1 peptides (MYM-V111 or virosome-P1) and the virosomes with rgp41 (MYM-V112 or virosome-rgp41). The manufacturing process of these influenza-derived virosomes remains as before^22,33^, except that trehalose as new selected excipient is added during the in vitro virosome formation process (Fig. 1a). Trehalose is therefore encapsulated inside the virosomes and is present on the outside of the particles in the final liquid virosomes at the same concentration (50 mg/mL). The addition of trehalose contributes to preserve virosome integrity during the downstream processes to manufacture solid dosage forms. Other excipients from the Generally Recognized as Safe list from the Federal Food and Drug Administration were added to the MYM-V202 bulk solution, such as alginate to facilitate mucoadhesion of the spray-dried nasal powder or fish gelatin acting as a support matrix for the lyophilized sublingual tablets. These specific additions helped to achieve suitable visual and physical attributes and properties according to specificities of each pilot line, which can be only briefly outlined in this manuscript due to proprietary information remaining as industrial know-how, with some complementary information described in the Supplementary Results.

The presence of the new excipients required for each solid dosage did not provoke virosome aggregation during powder dissolution. Most of the virosomes from the population are detected as single particles, as visualized by AMNIS ImageStream that combines microscopy and flow cytometry (Fig. 1b, upper left panel) and by nanoparticle tracking analysis (NTA) (Fig. 1c, one major single peak). The particle distribution of the reconstituted nasal, oral, and sublingual powders obtained by NTA confirmed that the population distribution and mean virosome diameter size (ranging from 96 to 109 nm) remained comparable to the liquid virosome (diameter 116 nm) and remained stable for several hours at room temperature.

As opposed to the liquid formulation, with most of the virosomes existing as single particles, reconstituted powders have a slight increase in the number of virosomes that tend to form small clusters around 150–200 and 200–300 nm (Fig. 1b, c) constituted by 2 or 3 particles, but they generally represent a minor population (<15%). With the sublingual tablets, this proportion of small virosome clusters is more marked as a clear distinct population. Although rare, larger aggregates >300 nm can be visualized by NTA (Fig. 1c) for all formulations, which can be more frequent in the sublingual formulation. This is presumably due to the presence of the hydrolyzed gelatin excipient that may potentially agglomerate after lyophilization and reconstitution, which could favor virosome clustering and trapping. At this stage, the presence of some damaged virosomes after downstream processing that may also contribute to small cluster formation or aggregates cannot be excluded.

### In vitro evaluation of virosomes

Prior to moving to in vivo experiments with the new solid dosage forms, two early immunological events were monitored in vitro with antigen-presenting cells (APC): (i) absence of acute cell toxicity and (ii) no impact on cell ability to up-take virosome particles. This is to determine if there would be a potential risk of failure in inducing immune responses in animals. Although the selected excipients are not expected to have any acute toxicity effect because they have been evaluated extensively in diverse studies, certain excipients such as the trehalose may represent >70% of the vaccine composition for the nasal powder, and if applied to nasal tissues, although unlikely, an impact on APCs cannot be excluded. Considering the difficulty to have access to human sublingual, nasal, and ileum mucosa cell culture systems with APCs, the alternative was to perform these studies on human dendritic cells (DCs) in cell culture.

**Fig. 2:**
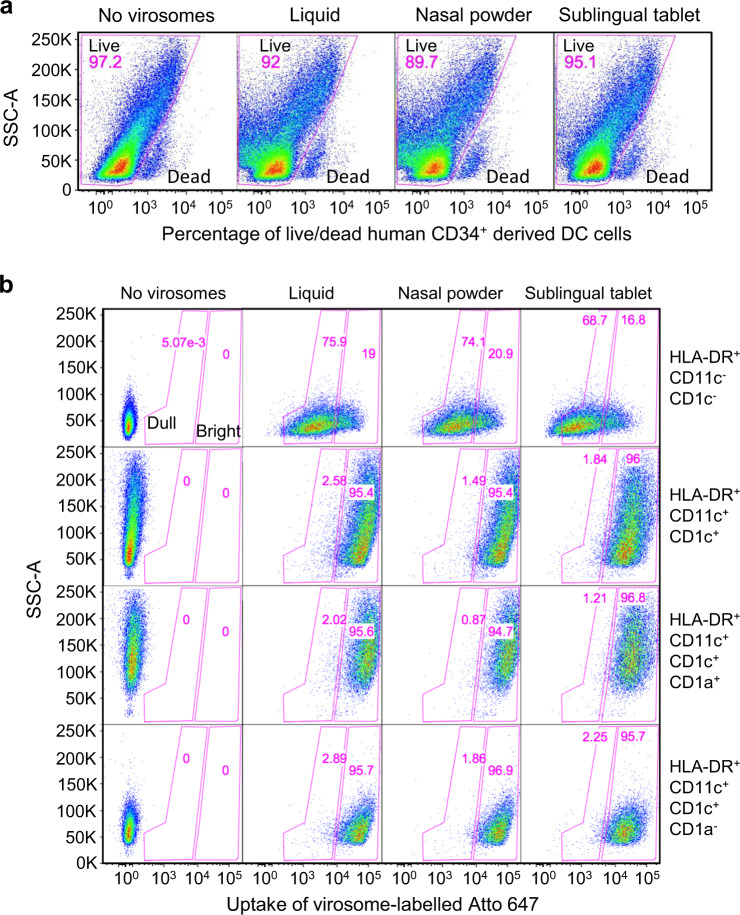
In vitro virosome toxicity and uptake by antigen-presenting cells. **a** Human CD34^+^-derived cells in culture were exposed to the various virosome formulations for 1 hour and cells were then stained with LIVE/DEAD dye to determine the percentage of dead cells and cells alive. **b** Virosomes were labeled with a stable tracer fluorescent lipid labeled with Atto 647 and their uptake after 1 hour by various APC subpopulations defined by markers HLA-DR, CD11c, CD1c, and CD1a were monitored by cytometry, gating on dull and bright virosome signals in pink gates (gating strategy described in Supplementary Methods Fig. 3). Cells that are HLA-DR^+^CD11c^−^ are not differentiated into dendritic cells and they represent the majority of the population. Cells that are HLA-DR^+^CD11c^+^CD1c^+^CD1a^+^ have a phenotype similar to Langerhans cells, and cells that are HLA-DR^+^CD11c^+^CD1c^+^CD1a^−^ are more similar to dermal DC. The stronger is the fluorescent Atto 647 signal, the more virosome uptake took place. The percentage of Atto 647 positive cells is comparable between cells exposed to the starting liquid formulation and the various solid vaccine dosage forms, suggesting that excipients did not interfere with early virosome uptake by APC. Data are from a representative experiment.

Fluorescent Atto-647 labeled placebo virosomes containing only influenza HA were formulated as liquid virosomes that remained stable and fluorescent after downstream processing into nasal, oral, and sublingual solid forms. Human CD34^+^-derived DCs as professional APCs were incubated for 1 h with virosomes-Atto 647 from reconstituted nasal, oral, or sublingual formulations (Fig. 2a). Although the incubation time is short with DC and may not detect slow toxicity effects that could lead to program cell death by apoptosis or pyroptosis^65^, a longer incubation was not considered because in vivo, those excipients in solution would be rapidly diluted within minutes by the local fluid at the administration site, further reducing the risk of any potential toxic effect on APCs. The LIVE/DEAD dye added to the cell culture indicated no significant acute toxicity on cells, as similar percentages of living cells (90–95%) were observed in the presence of virosomes.

Virosomes-Atto 647 uptake by these APCs were then evaluated (Fig. 2b). Four key different APC subpopulations were generated during the cell culture of human CD34^+^ that could be distinguished by gating strategies by flow cytometry. First on the overall general SSC/HLA-DR^+^ population, then on the CD11c/CD1c markers for visualizing the double negative CD11c^−^/CD1c^−^ and double positive CD11c^+^/CD1c^+^ subpopulations, the latter one for identifying the CD11c^+^CD1c^+^/CD1a^−^ and CD11c^+^CD1c^+^/CD1a^+^ subpopulations. Figure 2b shows that among each subset of APCs there was no difference in virosomes-Atto 647 uptake between the various vaccine formulations but a lower signal in HLA-DR^+^/CD11c^−^/CD1c^−^ cells was noticed.

### In vivo virosome evaluation

The new galenic formulations were developed for direct mucosal administration to humans. At first glance, testing the impact of excipients on the vaccine immunogenicity after mucosal delivery on small animals is attractive. However, considerable inter-species anatomical differences at the mucosal level exist, particularly between human and small animals, such as the mucosal tissue composition and thickness, pH, and transit time. These factors all affect the local residence time of the vaccine at the mucosa and the virosome migration to the lymph nodes. Considering these factors, data interpretation is complicated and may not be directly translated to human. Understanding the mucosal vaccination efficacy in a more relevant animal model is important and there is an ongoing independent study on non-human primates supported by the National Institutes of Health (NIH), prior moving into human trials.

**Fig. 3:**
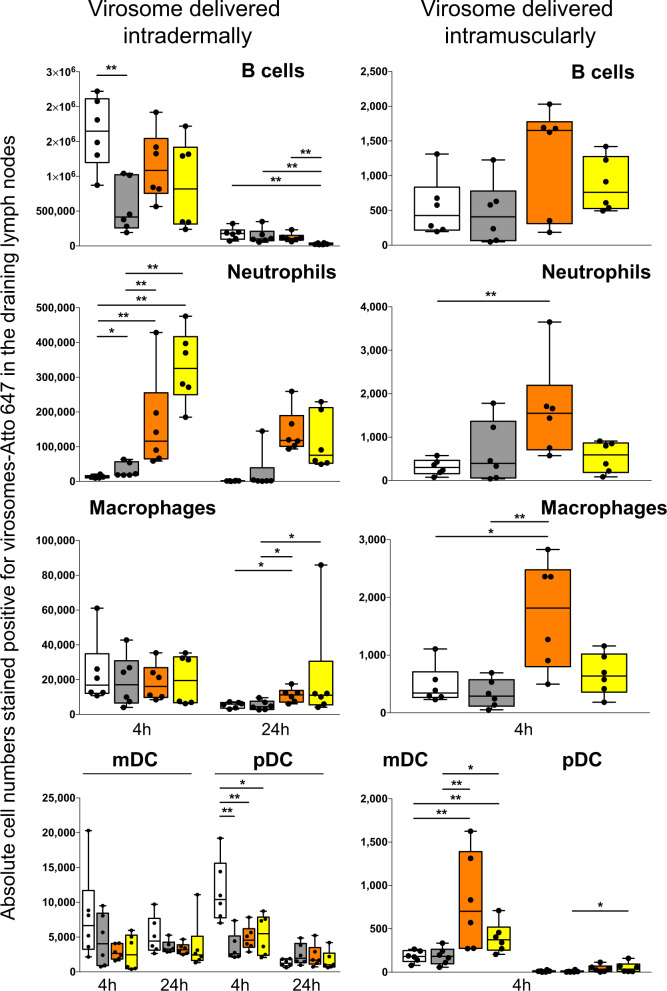
Impact of excipients on in vivo migration of virosomes from liquid and solid dosage forms. The absolute number of virosome positive Atto 647 in B cells, neutrophils, macrophages, myeloid DCs (mDCs), and plasmacytoid DCs (pDCs) present in two draining lymph nodes of mice after 4 or 24 hours following intradermal injection (panels on the left column) or after 4 hours following intramuscular injection (panels on the right column). Cell subpopulations were defined (gating strategy described in Supplementary Methods Fig. 4) by the presence or absence of various cell surface antigens (markers): B cells (B220^+^CD11C^−^Ly6C^−^), neutrophils (CD11b^+^Ly6C^+^Ly6G^+^), macrophages (I-Ab^+^CD11b^+^F4/80^+^Ly6C^Lo^Ly6G^−^CD11c^−^), myeloid DCs (I-Ab^+^CD11b^+^Ly6C^Lo^Ly6G^+^CD11c^+^), plasmacytoid DCs (I-Ab^+^B220^+^Ly6C^+^CD11c^+^). Solid dosage forms were dissolved in water prior injection and similar hemagglutinin dose were administered. Liquid virosome (white bars) prior downstream processing, sublingual tablet (gray bars), oral powder (orange bars), and nasal powder (yellow bars). Each vaccine group had six mice, and the corresponding data for each animal is displayed for showing distribution within box-and-whisker plots, with the means and standard deviations for the measured cell subpopulations in the lymph nodes. Statistical analyses used the Mann–Whitney *U* test and statistical significances between mice groups that received different virosome formulations are indicated: *p < 0.05, **p < 0.01.

Due to inter-species limitations, results presented in this manuscript focus on the delivery of the same volume of reconstituted vaccine powders by either intramuscular, subcutaneous, or intradermal (ID) route. Note that liquid virosomes are generally administered intramuscularly to humans during vaccination, although unadjuvanted virosome-based vaccines against hepatitis A and seasonal influenza were shown to be also immunogenic and tolerated in humans, following intradermal vaccinations^24,66^. Vaccine injection in the intradermal immune-rich environment is thought to enhance the antigen immunogenicity and may contribute to antigen dose sparing for cost saving. However, ID virosome vaccination also led to higher incidence rate of solicited local adverse events (e.g., erythema and induration), indicating that vaccine formulation must be improved. Both IM and ID routes were attractive and tested for monitoring the virosome migration to the draining lymph nodes (Fig. 3). Comparing liquid virosomes as the reference material to solid forms represented the last experiment before conducting immunogenicity study in animals. Mice received a single intramuscular or intradermal injection of virosomes-Atto 647 from liquid or reconstituted nasal, oral, and sublingual powder. Draining lymph nodes were then collected after 4 and 24 hours to isolate cells and enumerate the cell subpopulations fluorescent for virosomes-Atto 647 (Fig. 3).

Although the different virosome formulations led to various numbers of Atto 647 positive cells, overall, none prevented virosomes from reaching the draining lymph nodes. Four hours after IM injection, the absolute number was found to range from about 200 to 1600 positive cells among the different subpopulations, except for the plasmacytoid DCs with only about 10–50 positive cells (Fig. 3, right panels). Although some statistical differences were observed between vaccine formulations, considering the limited positive cell numbers and that these cells were barely detectable after 24 hours (data not shown), it is unlikely that such differences could impact the antibody response. However, 4 hours after ID injection, the absolute cell number was at least 100-fold higher for B cells, neutrophils, and pDCs, and at least 10-fold higher for macrophages and myeloid DCs, respective to IM route (Fig. 3, left panels), and 24 hours later the cell numbers were still higher than 4 hours post-intramuscular injection. For a given cell subpopulation, there were also statistical differences between the vaccine formulations.

The impact on the immune outcomes due to a higher number of immune cells positive for virosomes Atto 647 in the lymph nodes after ID injection, respective to IM, was not investigated because those vaccines are intended for mucosal vaccination. Meanwhile, we can postulate that ID virosome injection is more likely to induce a stronger antibody response, as compared to IM injection, as already reported^24^. It is less certain that the induced antibody response after ID injection would significantly differ among the various virosome formulations. Furthermore, neutrophil recruitment was very low with liquid and sublingual virosomes, as compared to nasal and oral formulations, which could be due to the high trehalose content in these two formulations, this would require more investigation. Overall, this suggests that virosomes could either migrate as free form particles for reaching most likely the subcapsular lymph node region rich in macrophages, and/or by active cell transport, contributing to antigen accumulation into the lymph nodes that is essential for triggering the immune response^67–72^. Based on previous studies, labeled virosomes remain stable inside cytoplasmic vesicles (e.g., endosomes) prior being disassembled after few hours. Therefore, the fluorescent signal, particularly at 24 hours, could come either from intact virosomes or released Atto 647 inside cell vesicles.

**Fig. 4:**
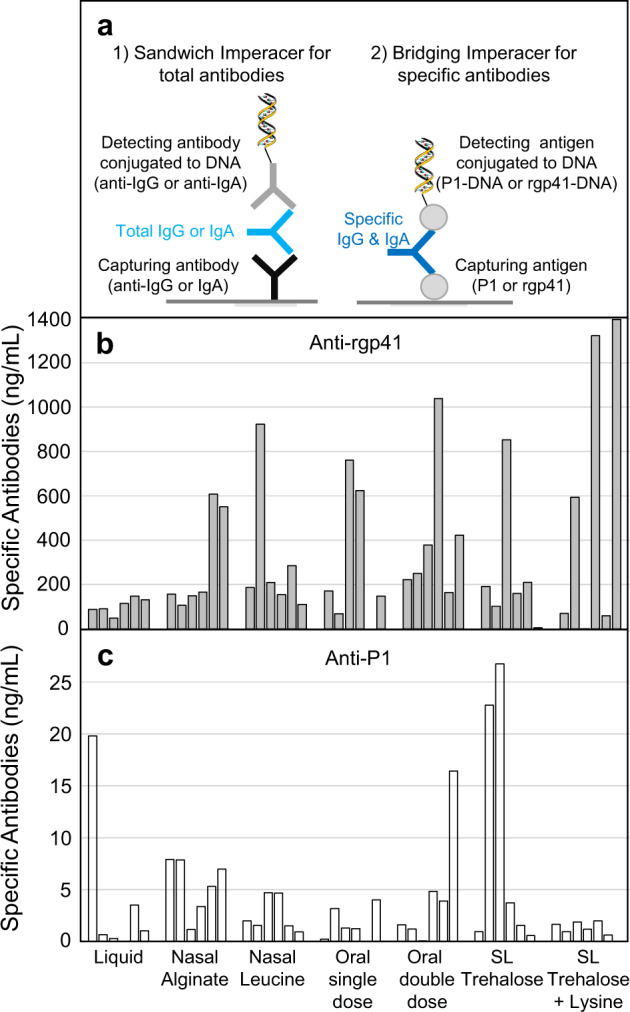
Vaccine-induced serum antibodies in the presence of various excipients. Early development research grade lots (not optimized yet) of the virosomal vaccine MYM-V202 under nasal and oral powder, and sublingual (SL) tablets were reconstituted in water and were administered subcutaneously to rats at day 1 and day 28 to determine if excipients of each new solid dosage form were affecting the antibody response. Each final serum at day 42 was analyzed by the Imperacer assays developed during MACIVIVA project, as illustrated in (**a**). Data of each animal is shown for better appreciating of the inter-individual variation often observed with suboptimal formulations. Approximate net serum (pre-immune deducted) anti-rgp41 antibody concentrations detected at day 42 (**b**), and anti-P1 antibody concentrations detected at day 42 (**c**). Data are from a representative Imperacer assay experiment.

For immunogenicity studies, the IM route could have been maintained for consistency with the previous in vivo study on virosome migration but the SC route was chosen as a compromise between IM and ID routes. With SC injections, potential side effects or local intolerance at the injection site can be detected, while it may not be revealed by IM injection. For the ID route, with TLR7/8 agonist as an adjuvant on the virosomes, the risk of strong local intolerance for each vaccination was thought to be too high, and this could impact more the immune response, as compared to SC route. Figure 4 presents data from the reconstituted solid forms (nasal, oral, sublingual) administered subcutaneously to rats. Prior to administration, the powder quantity (mg) required to achieve similar antigen dose as the liquid control was dissolved and reconstituted into water. In Fig. 4b, c, the quantification of the anti-rgp41 and anti-P1 antibodies are respectively shown, with the starting liquid virosomes prior to downstream processing used as the reference material. Overall, the antibody response induced by solid dosages was at least equal to the liquid dose control. Note that sublingual tablets containing lysine for improving the lyophilization process of sublingual tablets did not improve the P1 immunogenicity, respective to tablets without lysine. Therefore, at very early stage, sublingual tablets with lysine were no longer considered.

GMP manufacturing processes were successfully established for the synthetic P1 peptide and the recombinant rgp41 protein. According to the stability studies, both frozen APIs remain stable for at least 2 years (Supplementary Fig. 1). With these GMP lots of APIs, the liquid GMP HIV-1 vaccine MYM-V202 was manufactured and then distributed to partners for spray drying and lyophilization.

### Stability and immunogenicity of the new virosome powder forms

**Fig. 5:**
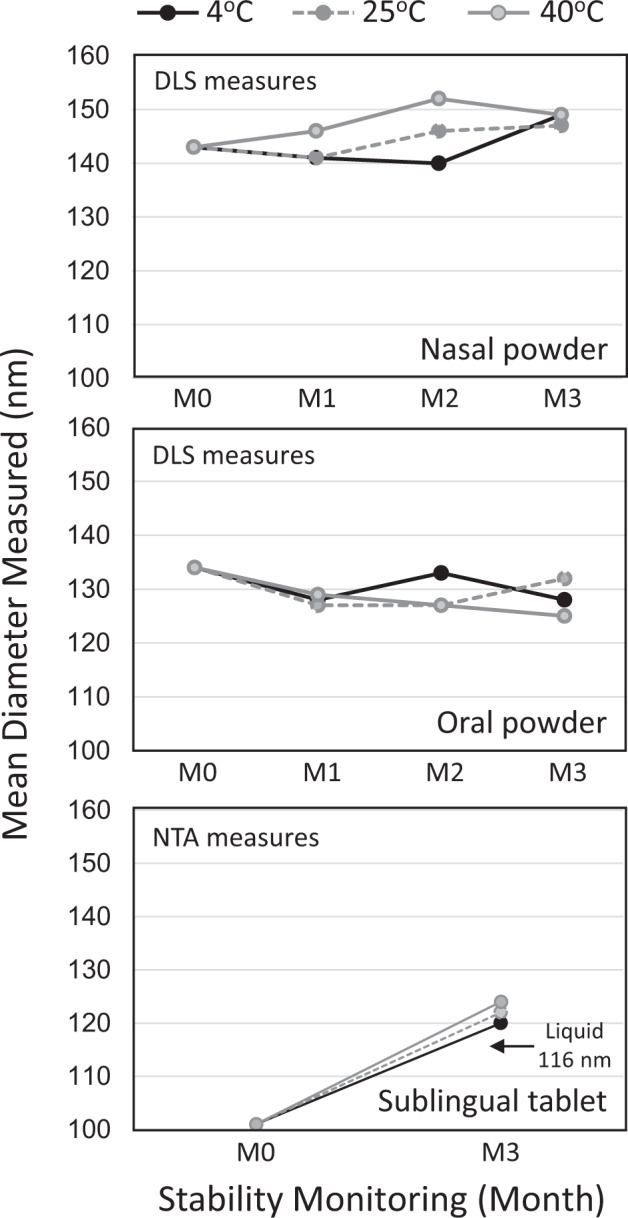
Stability of the virosome particle size after storage of the vaccine solid forms under various environmental conditions. A 3 months stability study was performed on the various solid vaccine forms (nasal and oral powders, sublingual tablets) stored under three different temperatures and relative humidity (RH) conditions: 4 °C (black line), 25 °C/65% RH (gray dot line), and 40 °C/75% RH (gray line). At each indicated month time point (M0–M3), samples were reconstituted with water and the mean virosome particle size (nm) was determined. Due to specific excipient interference during particle size analysis and different equipment available at different manufacturing sites, different methods were selected for particle analysis during stability study: DLS for nasal and oral powder and NTA for sublingual tablets. Note that for the sublingual virosomes, the mean particle size (101 nm) is about 10% smaller after lyophilization at M0, respective to the liquid virosomes (116 nm), but it is closer to the starting size after 3 months storage (120 nm at 4 °C and 124 nm at 40 °C). The higher residual moisture content in sublingual tablets (about 4%) and nasal powder (about 3%), respective to the oral powder (about 2.5%) may have contributed to bring back the virosome size closer to the original liquid virosome size over time. Data shown are from representative DLS and NTA measures.

**Fig. 6:**
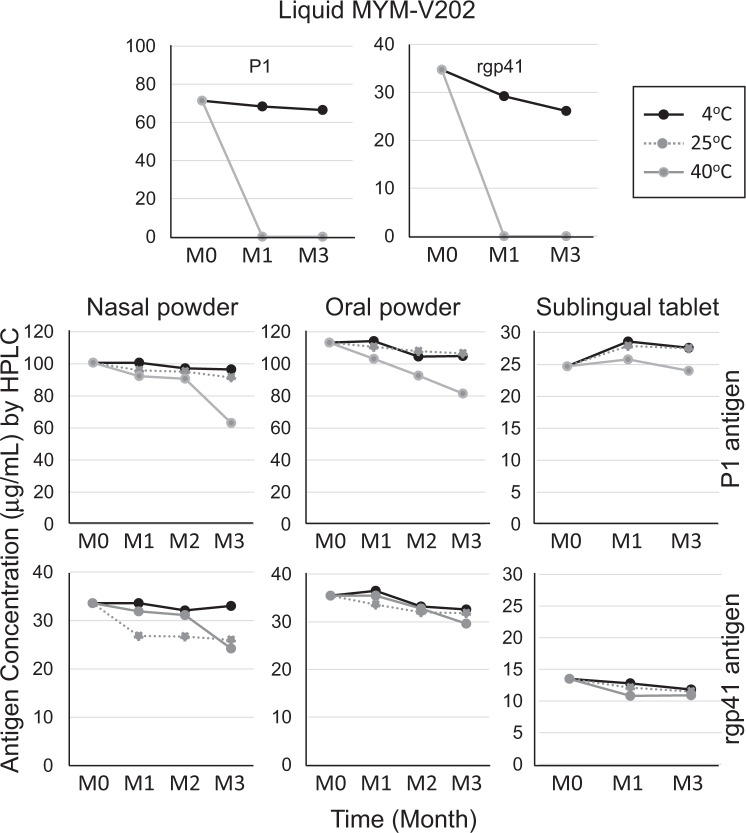
Antigen concentrations in the various vaccine forms exposed to different environmental conditions. The liquid adjuvanted vaccine formulation MYM-V202 containing both P1 and rgp41 antigens served as reference material for comparison to the solid vaccine dosage form for nasal, oral, and sublingual delivery. A 3 months stability study was performed on the various liquid and solid vaccine forms stored under three different temperatures and relative humidity (RH) conditions: 4 °C (black line), 25 °C/65% RH (gray dot line, not done for the liquid form), and 40 °C/75% RH (gray line). P1 and rgp41 antigens were previously shown to be temperature sensitive, which is confirmed again here in the first two upper panels, showing rapid P1 and rgp41 modifications at 40 °C, as compared to the liquid vaccine stored under the recommended temperature at 4 °C. For solid vaccine forms, at each indicated month time point (M0, M1, M2, or M3), samples were reconstituted with water and analyzed by HPLC for the P1 and rgp41 content. Note that chemical modifications such as oxidation or deamidation on antigens are the main reasons to the observed lower antigen concentration, as antigen degradation could not be reported by native immunoblot. Data shown are from representative HPLC measures.

Small non-GMP lots for animal studies and a larger batch size of GMP grade solid dosages were manufactured during the last 6 months of the MACIVIVA project. Preliminary 3 months stability studies (Supplementary Fig. 2 for an overview of the study) were conducted in parallel to the immunogenicity study with vaccines stored under different environmental conditions. Particle size measurements by NTA and dynamic light scattering (DLS) indicated that the virosomes remained stable at 4 °C, 25 °C, and even when at 40 °C for 3 months (Fig. 5). Most importantly, we observed that in solid dosage forms, there was no significant degradation or chemical modifications of antigens P1 and rgp41 during the first 2 months, even when stored at 40 °C (Fig. 6). The antigen content measured by HPLC also showed very weak variation over time. As it was observed for the APIs, the 3M-052 adjuvant showed a similar stability profile as previously reported for a liposome-based vaccine^60^. After 3 months at 40 °C, a more pronounced decrease in the API contents was noticed, with a reduction varying from 5 to 30%, depending on the API and formulation, which was more pronounced for the nasal and oral powders.

**Fig. 7:**
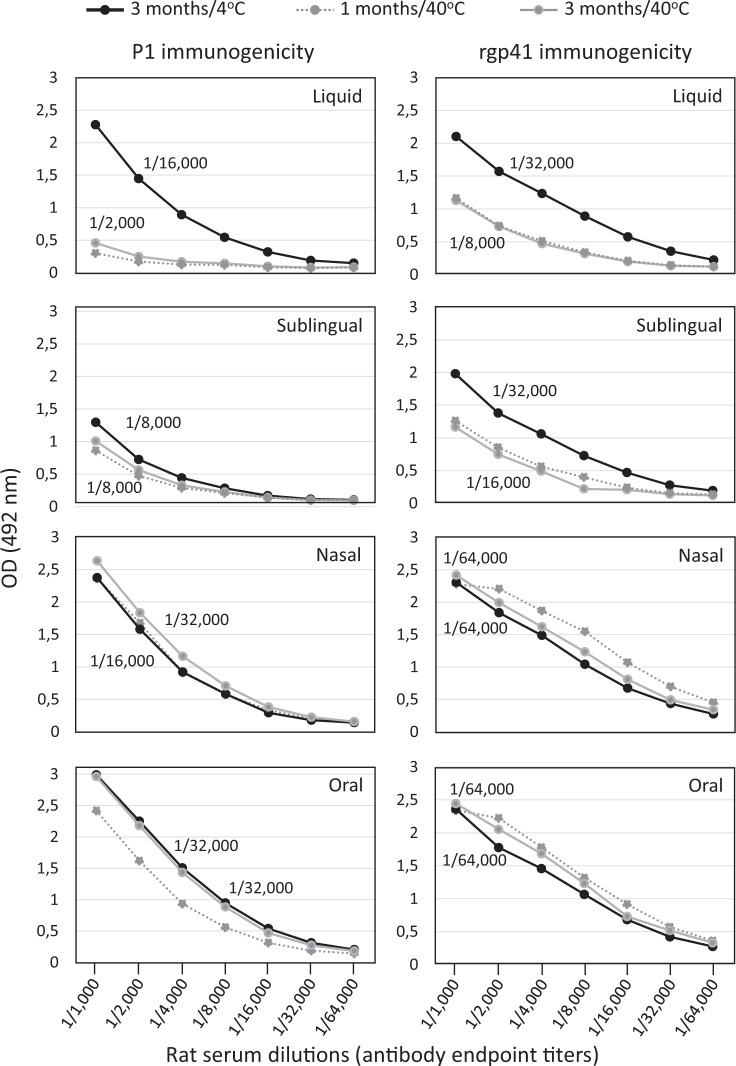
Immunogenicity of P1 and rgp41 from liquid and various solid vaccine dosage forms exposed to different temperatures. The liquid adjuvanted vaccine formulation MYM-V202 containing both P1 and rgp41 antigens were temperature sensitive and served as reference material for comparison with the immunogenicity of the solid vaccine forms with improved thermostability. Black line, vaccines stored 3 months at 2–8 °C; gray dot line, vaccines exposed 1 month at 40 °C; gray line, vaccines exposed 3 months at 40 °C. In each panel, the antibody endpoint titers (specific toward P1 or rgp41 antigen) of the serum pool of 10 rat sera are indicated, data are from a representative experiment. Endpoint titer against each antigen corresponds to the last serum dilution generating an optical density (OD) value >2-fold above the pre-immune background.

The new thermostable solid dosage forms have demonstrated to be physically and biochemically stable for at least 2 months at 40 °C/75% RH, when properly packed and stored. Figure 7 shows that all solid virosome forms of the HIV-1 vaccine candidate MYM-V202 exposed to 40 °C for 3 months (gray lines) had also retained their immunogenicity, as antibody endpoint titers were comparable to the corresponding solid formulation stored at 4 °C (black lines). This contrasts with the liquid form exposed to 40 °C with weaker P1 and rgp41 immunogenicity, as shown by reduced antibody endpoint titers. Note that for the sublingual tablets stored at 4 and 40 °C, the P1 immunogenicity appeared weaker, respective to the nasal, oral, and liquid forms.

**Fig. 8:**
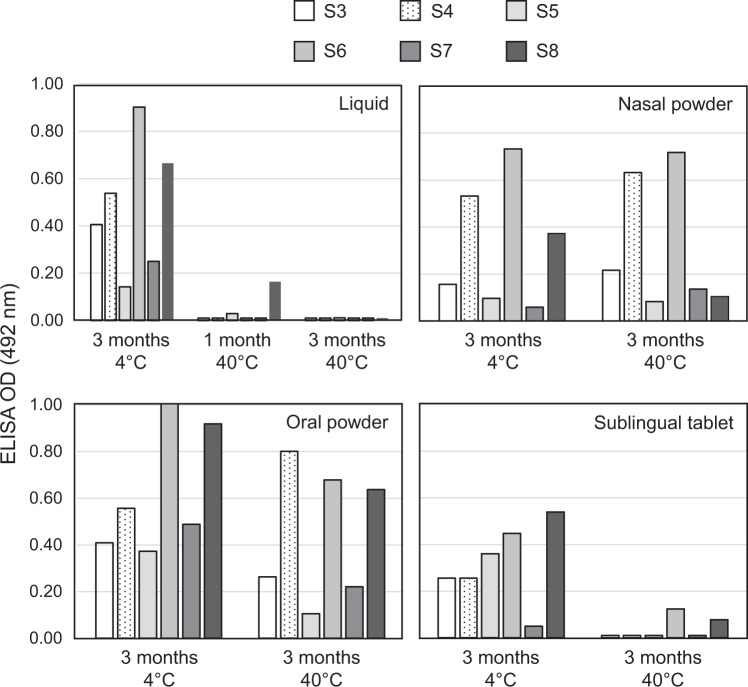
Key gp41 epitopes on vaccinal antigens are preserved in the new solid vaccine forms. The vaccine-induced antibody reactivity profile was tested toward six different gp41 biotinylated peptides (S3–S8) that were captured by pre-coated streptavidin ELISA plate. Rat serums from animal immunized with the liquid HIV-1 vaccine MYM-V202 stored at 4 °C serve as reference for determining the starting serum antibody reactivity toward the different gp41 peptides. Then, the liquid MYM-V202 was exposed at 40 °C for 1 and 3 months and used for immunizing animals, and the vaccine-induced serum antibodies were tested for their reactivity profile toward the selected peptides, and compared to the serums from animals immunized with the vaccine stored at 4 °C. Similarly, the nasal, oral, and sublingual solid vaccine forms were also stored at 4 °C or exposed at 40 °C for 3 months for evaluating the impact of heat exposure on antigen immunogenicity. As the antibody repertoire toward the P1 and rgp41 vaccinal antigens corresponds to the sum of various recognized epitopes, detecting the serum reactivity toward a single peptide/epitope is expected to be weak, justifying ELISA assays limited to dilutions from 1/200 to 1/1200. The data shown here correspond to the serum pool diluted 1/600 with pre-immune background removed, it corresponds to the dilution giving sufficient optical density (OD) signals among all peptides and conditions to determine if a given epitope was preserved intact, reduced or lost. OD values measured at 492 nm from representative ELISA tests are reported on the *Y*-axis.

Thus, the new GMP manufacturing processes and the elevated temperature exposure during storage had no significant impact on the overall vaccine immunogenicity (Figs. 4 and 7). However, this does not exclude the possibility that certain key protective epitopes were altered, compromising the vaccine bioactivity, while other epitopes playing no role in protection remained intact and contributed to the overall antibody endpoint titers. To investigate this further, ELISA epitope mapping was undertaken (Fig. 8) with short biotinylated peptides covering the core P1 and rgp41 epitopes (Table 1), most corresponding to those cited above (e.g., QARILAV, caveolin 2F5, 4E10, 10E8). Serum antibodies losing reactivity toward peptides S1, S2 that have no significant role in protection is a minor concern. However, losing reactivity against epitopes S3, S4, S5, S7, and S8 that may contribute significantly to protection, can greatly reduce the vaccine potency. Note that the proposed peptides can be recognized only by a small fraction of the entire antibody population, therefore weak signals were expected by this method.

**Table 1:** Biotinylated rgp41 peptides used for ELISA epitope mapping.

Antigen	Peptide	Epitope name	Length (aa)	Biotinylated gp41 peptide
gp41	S1	N-terminal	13	Biotin-MQARQLLSGIVQQ
	S2	Bacterial mimotope	13	Biotin-QQNNLLRAIEAQQ
	S3	HEPS	18	Biotin-GIKQLQARILAVERYLKD
	S4	Caveolin	18	SLEQIWNHTTWMEWDREI-K-Biotin
	S5	Transcytosis	16	EINNYTSLIHSLIEES-K-Biotin
gp41/P1	S6	5F3	15	SQTQQEKNEQELLEL-K-Biotin
P1	S7	2F5	17	Biotin-NEQELLELDKWASLWNW
P1	S8	4E10/10E8	17	Biotin-LWNWFNITNWLWYIKLS

These peptides are captured in streptavidin coated ELISA plates and used for evaluating the gp41 epitope reactivity profile of vaccine-induced serum antibodies in rat. Peptides have 13–18 amino acids (a.a.) in length and were derived from various gp41 regions of the HIV-1 HXB2 clade B strain, starting from the amino-terminus to the carboxyl-terminal end. Peptides S1 and S2 were designed for monitoring the level of vaccine-induced serum reactivity toward the gp41 N-terminal region and the bacterial mimotope present on the gp41. For peptides S3–S8, the sequences are based on reported gp41 epitopes toward which antibodies may harbor antiviral activities (caveolin, transcytosis), a region recognized by neutralizing antibodies present in HEPS or by the 2F5 and 4E10/10E8 neutralizing monoclonal antibodies (mAb) harboring cross-strain and cross-clade activities, and the non-neutralizing epitope recognized by the 5F3 monoclonal antibody.

### Antigen epitope preservation during downstream processing

The recombinant rgp41 used in the HIV-1 vaccine candidate has only 113 residues with limited potential epitopes and the protein structure is relatively simple, most likely made of two short helices. The known epitopes recognized by gp41 monoclonal antibodies with inhibitory activities are generally limited to very short peptides. The proposed peptides/epitopes for screening the antibody reactivity may either remain as linear peptide or potentially adopt a simple conformation structure to generate a conformational epitope.

The three solid vaccine forms stored at 4 °C also induced serum antibodies recognizing the S3–S8 peptides, when compared to the recognized profile of S3–S8 peptides by sera of animals immunized with the liquid vaccine stored at 4 °C, although sometimes showing higher or weaker signals (Fig. 8). When liquid virosomes MYM-V202 were exposed to 40 °C, most if not all epitopes were lost after only 1 month, with complete loss after 3 months (Fig. 8). This contrasts markedly with the nasal and oral powder exposed for 3 months at 40 °C where all peptides/epitopes were still detected by serum antibodies of the corresponding immunized animals. For sublingual tablets stored at 4 °C, we already observed a weaker signal by ELISA for most of the peptide S3–S8, as compared to the starting liquid formulation, and only two peptides could still be detected (S6/5F3 epitope and S8/4E10-10E8 epitope) after exposure to 40 °C for 3 months. This combined with slightly weaker antibody endpoint titers achieved with sublingual tablets (Fig. 7), and at this stage we cannot exclude the possibility that the fish gelatin excipient, which is an additional source of antigens in the new galenic sublingual formulation, could compete with P1 peptides. In that case, a higher antigen dose could be required for minimizing the competition with peptides derived from the hydrolyzed fish gelatin during antigen processing. Ideally, as a complement to epitope mapping, it would have been desirable to confirm that the vaccine protection efficacy after intravaginal challenge was retained by studying protection in non-human primates vaccinated by the mucosal route with these solid dosage forms. However, this was beyond MACIVIVA objectives but there is an ongoing study supported by the NIH, which is intended to look at the immunogenicity after intramuscular and intranasal vaccination, and monitor animal protection after intrarectal challenges.

## Discussion

We developed a “universal” GMP liquid virosome feed stock compatible with the three pilot lines: spray-dried nasal powder for loading into a dry powder nasal device, spray-dried oral powder for loading into an enteric-coated capsule, and lyophilized fast dissolving sublingual tablets. The final non-GMP and GMP virosomes in liquid and solid dosages were characterized by numerous bioanalytical methods (listed in Supplementary Methods). For example, the effects of physiological pH and temperature on the powder and tablet dissolution, time of dissolution, etc. Due to this broad complexity, in this article we just present the essential aspects, focusing on the virosome particle analysis and antigen content and immunological properties that are required for good vaccines.

Among the three solid dosage forms, overall, nasal and oral powder have best preserved the virosome population, followed by the sublingual tablets (Fig. 1c). Exposure to 40 °C over 3 months had no significant impact on the particle size, although the size increased for nasal and sublingual virosomes but only for reaching values similar to the starting liquid virosome. At this stage, we cannot exclude the possibility that some virosomes were damaged during downstream processing. Such virosome-derived materials forming clusters or aggregates may remain immunogenic and could still be captured and processed by the sublingual APCs, but certain epitopes could be exposed and presented differently.

The newly developed thermostable HIV-1 solid vaccine forms had no significant impact on the antibody response and had no or limited effect on preventing access to antigenic epitopes, as comparable serum antibody levels were measured by the Imperacer assays (Fig. 4) and epitope specificities by ELISA (Fig. 8) were observed, when compared to the liquid formulation. When exposed to temperatures outside of the recommended storage condition of 2–8 °C, solid vaccine forms maintained their antigen content (Fig. 6) with limited impact on the immunogenicity of the vaccinal antigens, based on antibody endpoint titers toward P1 and rgp41 (Fig. 7) and the preservation of key antigenic epitopes (Fig. 8).

No significant antigen degradation occurred during the first 2 months at 40 °C, while at month 3, the nasal and oral powder had a more significant APIs reduction. Note that by native immunoblot (similar to Western blot), there was no evidence of degradation, as the antigen content remained unchanged, and the observed content reduction was detected only by HPLC methods that were sensitive enough to detect subtle chemical modifications on some residues that affect the API elution profile, resulting in lower API content. Subsequent investigations have demonstrated that the integrity of the packaging was compromised at high temperature for nasal and oral powder, leading to a gradual increase of the moisture content in the powder, likely favoring API chemical modification. Since that first study, the sealing and packaging processes were improved for the GMP manufacturing process and the moisture content is maintained low and stable. According to the NTA analysis, the virosome particles and population from solid forms were also found to be stable during 1-week exposure to <−15 °C (data not shown) which mimicked accidental freezing conditions susceptible to take place during shipment, which would usually destroy eVLPs.

ELISA epitope mapping showed a reduced or increased signal for certain peptides, respective to the liquid vaccine reference material. This variation is within the assay sensitivity and accuracy limits and may not be significant. As there is no loss of epitopes, it suggests that the three vaccines from thermostable solid forms have maintained all the initial epitopes during downstream processing. The development of new downstream processing pilots maintaining key protective epitopes was fundamental to preserve the vaccine potency. All the above strongly suggests that vaccination with the solid forms of the virosomal HIV-1 candidate vaccine should elicit relevant protective antibodies, even if the product would have been stored accidentally for few days or weeks at 40 °C or frozen during shipment.

In summary, the new developed thermostable HIV-1 solid vaccine forms have preserved most of the lipid-based virosome structure and the antigens with their key epitopes, and the vaccine immunogenicity was retained. These new “ready to use and all-in-one” products with vaccinal antigens and adjuvants located on the same particle better support specific immune activation and further improve the vaccine safety and tolerance. These solid dosages circumvent the need of reconstitution prior to administration, and they can be needle-free administered directly through nasal and oral mucosa. Administering the same HIV-1 gp41 virosomal vaccine through two distinct mucosal routes (e.g., nasal and sublingual) may potentially elicit a broader and more robust genital and intestinal immune response in both genders, and could be also employed in a prime-boost approach with another HIV-1 vaccine based on different antigens expressed by other technologies such viral vectors.

The MACIVIVA project has addressed one of the major challenges in the vaccine field that is the cold chain dependence of vaccines during shipment, distribution, and short-term storage. The new solid forms of the virosomal HIV-1 vaccine complies with the WHO recommendations with demonstrated short-term stability outside of the recommended storage temperature. Such vaccines with improved stability outside the cold chain will contribute to reduce vaccine loss.

## Methods

### Liquid virosome manufacturing

HXB2 clade B HIV-1 gp41-derived antigens used in this manuscript were previously described^22,33,47^: The synthetic P1 modified lipopeptide of 38 residues (sequence 649–684 followed by SC residues) was produced by Bachem AG (Bubendorf, Switzerland) as research grade and pharmaceutical (GMP) grade material. Research grade and GMP grade lots of the rgp41 with 115 residues (540–664 with a deletion of 25 amino acids from 593 to 618, plus a C-terminal His-tag for purification followed by a free cysteine) were expressed in *Escherichia coli* and purified as trimers under non-denaturing conditions by PX’Therapeutics (Grenoble, France). Lipidation of the C-terminal cysteine to 1,2-dipalmitoyl-sn-glycero-3-phosphoethanolamine-N-[4-(p-maleinimidomethyl)cyclohexane-carboxamide] (N-MCC-DPPE, Corden Pharma, Liestal, Switzerland) allowed antigen anchorage into the virosome lipid membrane produced under liquid form, as previously described^22^. The 3M-052 TLR7/8 adjuvant (3M Company, St. Paul, USA) was dissolved in 100 mM octaethylene glycol monododecyl ether (OEG, Sigma, Buchs, Switzerland) prepared in HN buffer (50 mM HEPES, pH 7.4, 142 mM NaCl) and added to the virosome excipients and antigen mixture during manufacturing. The final GMP liquid virosome MYM-V202 for downstream processing into solid powder forms contained 40 μg/mL of hemagglutinin (HA), 120 μg/mL P1, 70 μg/mL rgp41, 40 μg/mL 3M-052, and was supplied in HN buffer pH 7.4 with 50 mg/mL Trehalose SG (Hayashibara Co., Okayama, Japan). Fluorescent placebo virosomes for in vitro studies were produced by inserting the 1,2-dioleoyl-sn-glycero-3-phosphatidylethanolamine (DOPE, Merck & Cie, Schaffhausen, Switzerland) conjugated to the Atto 647 dye (DOPE-Atto 647) into the virosomal membrane. Quality controls were conducted with appropriate RP-HPLC methods for determining the concentration (μg/mL) of P1, rgp41, and 3M-052, Single Radial Immunodiffusion Assay for HA concentration (μg/mL), turbidimetric chromogenic assay with Limulus amebocyte lysate for endotoxin quantification (EU/mL). NTA for the virosome particle size was performed on a Malvern NS300 instrument. DLS for determining the virosome population homogeneity based on the polydispersity index was performed on a Malvern Zetasizer Nano S. Note that virosomes from liquid and reconstituted powders were also labeled with the Dil lipophilic tracer (1,1′-dioctadecyl-3,3,3′,3′-tetramethylindocarbocyanine perchlorate, Sigma, Buchs, Switzerland) just prior acquisition with Amnis® ImageStream® XMark II (magnification ×60) to visualize single fluorescent virosome particles, clusters, and aggregates. Microbiological quality was determined according to E.P. section 5.1.4. The absence of specific microorganisms was demonstrated according to E.P. section 2.6.13—*Pseudomonas aeruginosa* and *Staphylococcus aureus*. Non-GMP batch sizes were the equivalence of 100–500 vaccine doses, GMP batch had the equivalence 1 L, representing about 1500 liquid vaccine doses.

### Spray drying of virosomes for nasal and oral powder

Excipients were added to the GMP virosome feed stock solution for spray drying. The nasal powder formulation was obtained by adding trehalose and sodium alginate (mucoadhesive excipient) to the liquid virosomes MYM-V202 at an excipient loading to achieve 77 and 8% w/w, respectively. The oral powder formulation was obtained by adding trehalose 87% w/w. During spray drying the outlet temperature was set at 60 °C, using an inlet temperature of 85–90 °C. Final bulk powders were overlaid with nitrogen gas and stored at 2–8 °C, double bagged in foil pouches for light protection. Following analysis, glass vials and Aptar dry powder nasal devices were loaded with various amount of powder to fit the need of the animal and stability studies, then closed and sealed into double-bagged foil pouches for light and moisture protection. Depending on the need, samples were stored under various temperature and relative humidity (RH) conditions: 4 °C, 25 °C/60% RH, and/or 40 °C/75% RH. Each mg of GMP nasal and oral powder contained 0.50 μg HA, 1.5 μg P1, 0.83 μg rgp41, and 0.50 μg 3M-052, in HN buffer pH 7.4. Powders were then evaluated by industrial analytical methods and standards in place at Upperton (Supplementary Tables 1 and 2) such for the powder particle size, moisture content, endotoxin level, or/and microbiological purity. Non-GMP batch sizes were the equivalence of 100–500 vaccine doses and for GMP batch the equivalence of 500 doses (enough for a Phase I trial).

### Lyophilization for sublingual tablets

A premixture of aqueous base matrix formulation containing mannitol (structure former) and fish gelatine (matrix former) at pH 7.4 was prepared. Trehalose was then added and mixed, followed by the addition of the liquid virosome formulation. This virosome matrix mixture was kept at 10–15 °C and dosed by weight (predetermined aliquots of 50 and 500 mg dosing fill weight) into preformed aluminum blister pockets. Once dosed, the aliquots were frozen at <−60 °C and then annealed at <−15 °C for <9 h. The frozen units were then freeze-dried using a two-step freeze-drying cycle (<−20 °C for <28 h followed by <15 °C for <22 h). The manufacturing conditions were optimized to preserve sufficient virosomes during subsequent freezing and freeze-drying with the required particle characteristics and maintaining the vaccine bioactivity. Prior dosing, the non-GMP and GMP liquid mixture of virosomes-base matrix solution contained 10 μg/mL HA, 30 μg/mL P1, 17 μg/mL rgp41, 10 μg/mL 3M-052, in HN buffer pH 7.4. After water sublimation during the freeze-drying step, the 50 mg (about 50 μL equivalence) or 500 mg (about 500 μL equivalence) dosed aliquots gave approximately 8 and 80 mg of lyophilized sublingual tablets respectively, of which an estimated amount of 3% of the lyophilized tablet was the virosome formulation content (expressed as dry matter). Sublingual tablets were evaluated by industrial analytical methods and standards in place at Catalent (Supplementary Tables 1 and 2) such as physical appearance, disintegration time, or endotoxin content. Non-GMP batch sizes were the equivalence of 100–500 vaccine doses and for GMP batch the equivalence of 2000 doses (enough for a Phase I trial).

### Stability study

After the liquid virosome MYM-V202 manufacture and quality controls as described above, it was processed downstream into sublingual tablets (MYM-V212), nasal powder (MYM-V222), and oral powder (MYM-V232). Aliquots of the nasal and oral powder were taken into sealed glass vials, wrapped and double bagged in foil pouches for light and moisture protection. Note that recently, after MACIVIVA project completion, stability was also conducted on Aptar nasal devices loaded with powder and similar results were obtained. Sealed aluminum blisters of sublingual tablets were directly placed into the storage room without additional packaging. Freshly prepared liquid and solid vaccine forms were split into two distinct lots: one for animal immunizations and one for quality controls. Lots were stored at 4 °C, 25 °C/60% RH, and 40 °C/75% for 1 and 3 months, then powder forms were stored at −20 °C and liquid form stored at 4 °C until analyses. Samples were analyzed by HPLC for vaccine content for P1, rgp41, and 3M-052, together with particle size analysis by NTA and measurement of the moisture content for solid vaccine dosages. Animals were immunized with vaccine samples kept at 4 °C after being exposed to various environmental conditions or analyzed as described above.

### In vitro and in vivo virosome uptake

Human cord blood CD34^+^ precursor cells were cultured one week with 50 ng/mL of GM-CSF and 5 ng/mL of IL-4 (from Miltenyi Biotec) for differentiation in DCs^73^. About one million CD34^+^-derived DCs were incubated for 1 h at 37 °C with 100 ng (based on HA content) of either placebo liquid virosomes or the reconstituted placebo nasal powder or sublingual tablet with sterile water. Cells were then washed twice with phosphate-buffered saline (PBS) and stained with standard method after blocking Fc receptor prior antibodies toward human antigens: HLA-DR (clone LN3 diluted 1/50; eBiosciences catalogue number 47995642), CD1a (clone HI149, diluted 1/10; BD Biosciences, catalogue number 555806), CD11c (clone B-ly6, diluted 1/50; BD Biosciences, catalogue number 562393), CD1c (clone L161, diluted 1/200, Biolegend, catalogue number 331515), LIVE/DEAD^®^ (diluted 1/1000; Molecular Probes, catalogue number L34957). Two independent experiments with mice (6–8-week-old C56/BL6) were performed (six mice per group) for monitoring the virosome migration in vivo. Mice received 10 μL (HA at 10 mg/mL) intramuscularly (IM) or intradermally (ID) of placebo virosomes-Atto 647 from liquid or reconstituted nasal, oral, and sublingual powder. After 4 and 24 h draining, lymph nodes were collected, processed, and stained as previously reported^74^: CD11b (clone M1/70 diluted 1/66; BD Biosciences, catalogue number 557657), CD11c (clone HL3, diluted 1/66; BD Biosciences, catalogue number 563735), Ly6G (clone 1A8, diluted 1/66; BD Biosciences, catalogue number 560603), Ly6C (clone AL-21, diluted 1/100; BD Biosciences, catalogue number 553104), I-Ab (clone AF6–120.1, diluted 1/66; BD Biosciences, catalogue number 562824), CD45R/B220 (clone RA3-6B2, diluted 1/66; BD Biosciences, catalogue number 552771), CD170/SiglecF (clone E50-2440, diluted 1/200; BD Biosciences, catalogue number 552126), NK1.1 (clone PK136, diluted 1/100; BD Biosciences, catalogue number 553165), CD86 (clone GL1, diluted 1/66; BD Biosciences, catalogue number 564200), F4/80 (clone BM8, diluted 1/66; eBioscience, catalogue number 25-4801-82). Cells were analyzed by BD LSR Fortessa flow cytometer, Diva and FlowJo software. Gating strategy is described in Supplementary Methods; Supplementary Figs. 3 and 4.

### Animal immunogenicity study

All animal studies were conducted in accordance with the requirements of the Institutional guidelines from preclinics GmbH (study 1 on rats) and Davids Biotechnologie GmbH (study 2 on rats) and national guidelines and legislation on animal experiments in Germany, care, health and welfare, and performed with qualified and trained personnel. Animal experiments in Germany were approved by the Animal Experiment Committee of the LAVG of the State of Brandenburg (for preclinics) and the Ethical Committee in Regensburg (for Davids Biotechnologie). Study no. 1: Male Wistar rats (*n* = 6 per group) were immunized at d0 and d28 by the by subcutaneous route. An adequate quantity of sublingual tablets, nasal, or oral powder (mg) was dissolved in sterile water for achieving the target concentration: About 5 μg of P1, 12 μg of rgp41, and 3 μg of 3M-052 TLR7/8 adjuvant in 0.1 mL. From each animal, pre-immune serum was collected at day 0 and immune serum at day 42 for quantification of rgp41-specific antibodies (ng/mL) via Imperacer bridge assay. Study no. 2: Wistar rats (*n* = 10 per group, 50% of each gender) were immunized at d0, d28, and d56. An adequate quantity of sublingual tablets, nasal, or oral powder (mg) was dissolved in sterile water for achieving the target concentration to be administered by subcutaneous route: About 3 μg of P1, 1.7 μg of rgp41, and 1 μg of 3M-052 TLR7/8 adjuvant in 0.1 mL. The liquid vaccine contained 3.9 μg of P1, 2.2 μg of rgp41, and 1.3 μg of 3M-052 TLR7/8 adjuvant in 0.1 mL. Pre-immune serums were collected at day 0 and immune serums at day 65 for determining the endpoint antibody titers of the serum pool and serum reactivity toward antigen-derived peptides for epitope mapping.

### Immuno-PCR Imperacer

Imperacer® combines the ELISA-based method with the qPCR technique to amplify the artificial DNA, conjugated to the detecting molecule^75–78^. Below the Imperacer method developed for MACIVIVA, some additional information and instructions can be obtained at Chimera, following a service request. DNA-labeled P1 and DNA-labeled rgp41 were used in bridging assays for quantification of specific IgG and IgA antibodies and DNA-labeled IgG anti-IgG or anti-IgA for quantification of total IgG and IgA antibodies in sandwich Imperacer assay. This method is very sensitive, specific, and species-independent, as it can detect a broad range of antibody concentrations of any isotype and from any animal origin.

Assay volume for both specific and total antibody detection was 30 µL/well done in duplicate. The following amount of a serum sample for a given time point that was required for preparing the dilutions for the assay: 11 µL for detecting specific anti-P1, 3 µL for detecting specific anti-gp41, <1 µL for detecting total IgG and IgA, respectively. Unlabeled P1 (1 µg/mL) or rgp41 (0.5 µg/mL) provided by Mymetics SA were diluted in coating buffer (Chimera Biotec, catalogue no. C-010) and coated on Imperacer® microplate modules (Chimera Biotec, catalogue no. C-001) for at least 16 h at 4 °C. The coated microplates were washed automatically (HydroFlex, Tecan) three times with buffer A (no detergent) at pH 7.35 (Chimera Biotec, catalogue no. C-011), blocked for preventing unspecific interaction (Chimera direct block, Chimera Biotec, catalogue no. C-013), followed by three washing steps with buffer B (with detergent) at pH 7.35 (Chimera Biotec, catalogue no. C-012), and subsequent incubation with pre-immune or immune samples. For P1-specific antibody detection, one volume of serum sample (11 µL) was mixed with 5 volumes (55 µL) of DNA-labeled P1 (conjugate “CHI P1”, Chimera Biotec, catalogue no. 11-313, diluted 1:300 in SDB5MAC, the sample dilution buffer, Chimera Biotec, catalogue no. C-093) for obtaining about 66 μL volume, which is sufficient for 2 × 30 μL per well for duplicate analysis. For rgp41-specific antibody detection, samples were first prediluted 1:12 (3 µL serum + 33 µL with PBS-Tween 20 0.05%, pH 7.33) and subsequently mixed 1:2 (33 µL + 33 µL) with a DNA-labeled gp41 (conjugate “CHI GP41”, Chimera Biotec, catalogue no. 11–292) diluted 1:300 in SDB6000 buffer (“Sample Dilution Buffer” Chimera Biotec, catalogue no. C-017), respectively. Following an incubation period of at least 16 h at 4 °C (for anti-P1) or 45 min at room temperature (for anti-gp41), plates were washed three times with buffer B, followed by two final washing steps with buffer A. PCR-Mastermix (Chimera Biotec, catalogue no. C-022: including DNA-label specific primers and real-time PCR probe; DNA label and primer sequence property of Chimera Biotec) were finally added to each well and the sealed plate was placed in a real-time PCR instrument (Chimera Biotec, catalogue no. 25-002) for signal generation. A bound antibody in the bridging assay connects its first single Fab part to the coated unlabeled antigen (“capture”), while another Fab part binds DNA-labeled P1 or rgp41 antigen acting as a “detector”. The DNA which is thereby immobilized due to the presence of specific antibodies was amplified during real-time PCR (50 cycles; each cycle = 12 s, 95 °C; 30 s, 50 °C; 30 s, 72 °C). As reference material, human anti-P1 2F5 mAb (Polyimmun Scientific, catalogue no. AB001) from 218.7 to 0.1 ng/mL, and human anti-gp41 mAb 5F3 (Polyimmun Scientific, catalogue no. AB010) from 1028 to 0.01 ng/mL were utilized for the preparation of a standard curve.

For IgG and IgA total antibody quantification, capture antibodies at 2 µg/mL in coating buffer (Chimera Biotec, catalogue no. C-010) were immobilized on the plate (goat anti-monkey IgG, Alpha Diagnostics, catalogue no. 70023; Goat anti-monkey IgA, KPL, catalogue no. 071-11-011). After at least 16 h coating at 4 °C, the microplate was washed (buffer A), blocked, and washed with buffer B as described above. The coated wells were subsequently incubated for 45 min at room temperature with diluted pre-immune or immune samples. Samples were diluted with PBS-Tween 20 0.05% at pH 7.33. Dilution was 1:30,000 for IgG detection and 1:300,000 for IgA detection, respectively. Following another three times washing step with buffer, antibody DNA detection conjugates were added to each well. For IgG detection, samples were incubated with DNA-labeled anti-IgG (CHI monkey IgG, Chimera Biotec, catalogue no. 11-324) diluted 1:300 in “Conjugate Dilution Buffer” (CDB, Chimera Biotec, catalogue no. C-020). For IgA detection, DNA-labeled anti-IgA was applied (CHI monkey IgA, Chimera Biotec, catalogue no. 11-323), also diluted 1:300 in CDB. Incubation was carried out for 45 min at room temperature. After a final washing step (three times with buffer B, followed by two times with buffer A, as above), PCR-Mastermix was added and PCR was carried out as described above. Real-time PCR signals were converted to approximate antibody concentrations (ng/mL) by analysis against a reference antibody curve. These antibody concentrations were provided only as indicative values.

### ELISA antibody endpoint titer

Maxisorp 96-well plate (Nunc-flat bottom) and Polysorp plates were respectively coated at 4 °C for 16 h with 0.1 mL of rgp41 or P1 peptide (2 μg/mL) prepared in PBS pH 7.4. Plates were washed 3 times with PBS with 0.05% (v/v) Tween 20 (PBST), then the blocking solution 1% (w/v) of bovine serum albumin (BSA) prepared in PBS with Tween (PBST) was added to each well and incubated 2 h at room temperature (RT). Plates were washed three times with PBST prior adding 0.1 mL per well of pre-immune serum diluted at 1/1000 or immune serum serial dilutions (from 1/1000 to 1/64,000) prepared in 0.1% BSA in PBST and incubated for 2 h at RT. Plates were washed three times with PBST and incubated for 2 h at RT with the goat anti-rat IgG-HRP diluted 1:4000 in 0.1% BSA in PBST. Plates were washed again before adding 0.1 mL of the colorimetric substrate o-phenylenediamine (OPD) and the reaction was stopped with 2 M H_2_SO_4_, followed by plate reading at 492 nm.

### ELISA epitope mapping

Streptavidin coated plates were used for capturing biotinylated peptides (5 μg/mL) S1–S8 (Pepscan, The Netherlands, see Table 1). About 0.1 mL per well (duplicate) of one of the eight different peptides prepared in PBS pH 7.4 was added, plates were incubated at room temperature for 2 h. Plates were washed three times with PBST prior adding 0.1 mL per well of pre-immune serum diluted at 1/600 or immune serum dilutions (1/300, 1/600, and 1/1200) prepared in 0.1% BSA PBST and incubated for 1 h at RT. Monoclonal antibodies 98.6, 5F3, 2F5, and 10E8 (at 0.25 μg/mL) served as positive control. Plates were washed three times with PBST prior adding either 0.1 mL of goat anti-rat IgG-HRP (Southern Biotech) or goat anti-human IgG (BioRad) in 0.1% BSA in PBST and incubated for 1 h at RT. After washing plates, each well received 0.1 mL of the OPD and the reaction was stopped with 2 M H_2_SO_4_, followed by plate reading at 492 nm.

### Reporting summary

Further information on research design is available in the Nature Research Reporting Summary linked to this article.

## Supplementary information

Supplementary Information

Reporting Summary

## Data Availability

Under reasonable request, the datasets generated during and/or analyzed during the current MACIVIVA study are available from the corresponding author. Due to proprietary information remaining as industrial know-how, the biological materials (API, adjuvant, virosomes, etc.) and protocols used for manufacturing that are described in the manuscript are restricted for each pilot line, as well as for the methods developed for characterizing the products.
