# Supplementary material for: New GMP manufacturing processes to obtain thermostable HIV-1 gp41 virosomes under solid forms for various mucosal vaccination routes
Source: NPJ Vaccines. 2020 May 18;5:41. doi: 10.1038/s41541-020-0190-9 (PMC7235025; doi:10.1038/s41541-020-0190-9)
Supplement: Supplementary file 1 — Supplementary Information [file 41541_2020_190_MOESM1_ESM.pdf]

Supplementary Introduction

Most of our R&D work conducted during the first two years of MACIVIVA project were not disclosed in this manuscript due to the large data set generated and industrial know-how behind this work from the different solid form manufacturers. Furthermore, several characteristics of each dosage form were evaluated, which has required analytical method implementations or the need to development new methods. In this Supplementary Information document, we are providing an overview of the methods used and some additional explanations to better understand the work undertaken during the manufacturing process development.

Supplementary Results

Process development

Since GMP manufacturing was limited to one GMP batch for each Pilot Line, batch consistency could not be evaluated. However, batch consistency was evaluated on different non-GMP lots and the quality control showed that the different non-GMP lots were always within the pre-defined specifications, such as the antigen and adjuvant contents, virosome particle size and distribution (presence of aggregates), physical attributes for the powder particle size (nasal and oral powder) and sublingual tablet appearance. The variation in the data sets between the different batches was below 10% for each parameter analyzed. Those data showing reproducibility for non-GMP lots are providing confidence that batch consistency will also be observed for the future different GMP lots.

The effects of physiological pH and temperature on the powder and tablet dissolution were also investigated to confirm that within seconds, the complete powder/tablet was dissolved/disintegrated under physiological mucosal conditions. The spray drying conditions and formulations were optimized for generating particles with most of the particle population having a volume mean diameter (VMD) > 9-10 µm, as particles with sizes < 5 µm are likely to be inhaled into the lung, while oral powder had a VMD of about 5 µm. The available commercial Aptar nasal device (Nasal Unidose Device from Aptar Pharma), as disposable single dose unit was tested and by gravimetric emitted dose we obtained > 98 % of intended dose delivered.

Once the oral powder was available, the next step was consisting in the selection of the appropriate enteric coated capsule that could be used for loading the oral powder. The capsule selection must be able to sustain the environmental conditions found in the human gastrointestinal tract during the transit journey from the mouth to the ileum as the final delivery site. During the screening process, the most promising capsule had for composition hydroxypropyl methylcellulose (HPMC) coated with an enteric polymer such as Eudragit (S100 and L100 in a 75:25 ratio). This capsule offered full resistance at pH 1.5.-3.0 over 2 hours (stomach pH conditions) and complete solubilization within minutes at pH 7.0 (human ileum pH condition), with full release of virosomes (no particle trapping) after capsule solubilization. Due to time constraints, the selected enteric coated capsule and powder loading process could not be implemented into GMP manufacturing. Therefore, the manuscript is presenting only the data obtained with the powder. Note that final capsule selection should also involve testing in non-human primates that have similar human gastrointestinal tract conditions. In this animal model,

it will be possible to confirm that virosomes are delivered properly to the ileum and local immune cells can uptake and process virosomes for eliciting systemic and mucosal antibody response.

Preliminary investigations on animals

We have conduced preliminary studies on different animal species, starting with the mucosal clearance rate upon virosome delivery at mucosal sites. Nasal powder delivered into rabbit nasal cavities showed a slow clearance rate (> 30 minutes), as expected due to the alginate as mucoadhesive excipient. Sublingual tablets in mini-pigs could locally remain about 15 minutes in contact with the sublingual tissue. This contrasts with the fast clearance of the liquid vaccine that may take a few seconds at the sublingual epithelium or < 5 minutes at the nasal cavities, as most liquids are mucosally delivered without adhesive properties.

Preliminary immunogenicity studies were conducted and those confirmed that nasal vaccination in rats and rabbits induced systemic, vaginal and rectal mucosal antibodies, based on detection by the Imperacer assays. It was postulated that the nasal powder form and the alginate as mucoadhesive excipient have most likely increased the retention time of virosomes, as observed in rabbits, which had contributed to improve the nasal vaccine immunogenicity, although the presence of the adjuvant had certainly played also a role. Sublingual tablets also induced mucosal antibodies at the vaginal and rectal levels of mini-pigs but serums antibodies were unexpectedly low, and we observed a high variability between animals, suggesting the need to further improve the sublingual formulation.

Inter-species mucosal barrier variation makes data interpretation difficult and it was subsequently decided to show in the manuscript only the immunogenicity data obtained after subcutaneous injection in rats and we focused on serum analysis. This approach was sufficient for obtaining qualitative data to confirm that the antigenicity and immunogenicity integrity of the newly developed solid dosage forms were preserved.

For evaluating mucosal vaccination with vaccines developed for human use, it was more adequate to conduct such investigations in non-human primates for obtaining representative data. Considering the high cost for such studies that could not be performed during the MACIVIVA project, complementary and independent studies are required at a later stage.

As the new HIV-1 adjuvanted virosomal vaccine under the powder form was very immunogenic in rabbits and rats, when delivered intranasally, this nasal vaccine is expected to be also immunogenic in NHPs and humans. There is an ongoing study supported by the NIH for investigating the vaccine-induced antibody response after intramuscular (liquid) and intranasal (powder) vaccination and determine if protection can be observed. The sublingual tablet immunogenicity in NHP is also of interest, particularly for improving the genital and intestinal antibody response, but this will be part of another investigation, priority was given to the nasal powder.

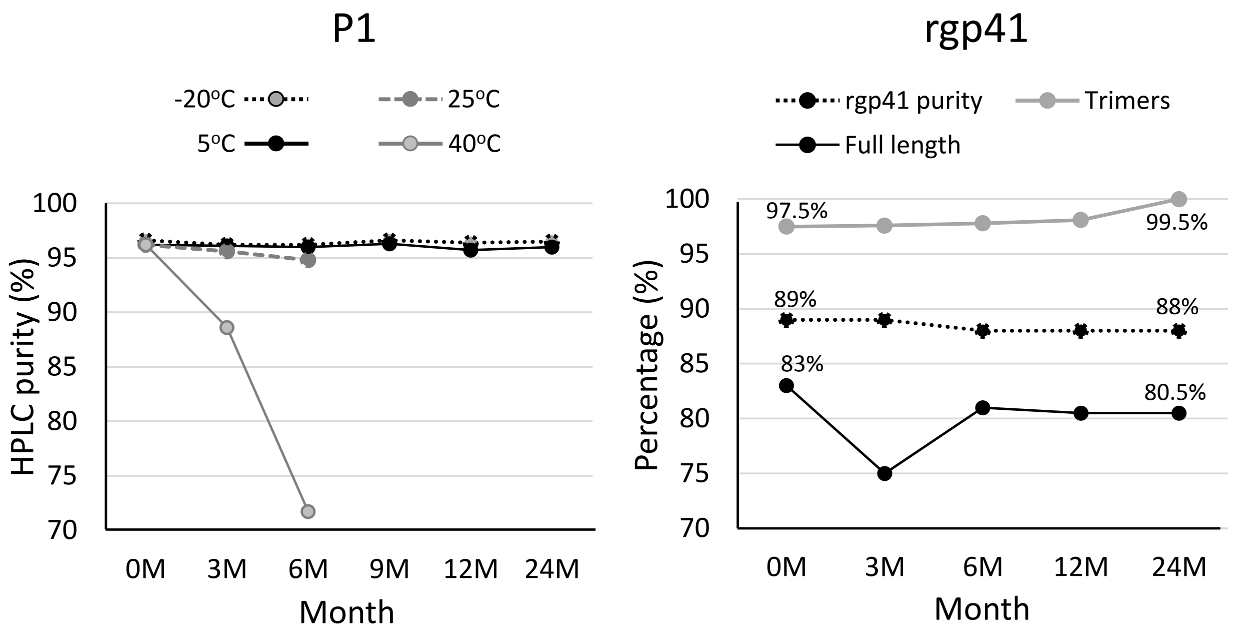

Supplementary Figure 1. Antigen and vaccine stability. Twenty four month stability data on the vaccinal antigens P1 (synthetic peptide) and rgp41 (recombinant protein produced in *E. coli*) stored under different environmental conditions. New and/or improved GMP manufacturing processes were successfully established for the synthetic P1 peptide and the recombinant rgp41 protein. This included the development of suitable bioanalytical methods for characterizing the APIs. Stability studies were conducted for both antigens over twenty months stored under various environmental conditions (according to ICH guidelines) to obtain first real-time data on the intrinsic stability of the test substances in an inert container. This provided information on typical degradation processes, on the expected future stability of the test substance, and on the suitability of the container closure system (data in supplementary information). Left panel: P1 stability determined by HPLC analysis on samples recovered at the indicated time point in month (M) after exposure to -20°C (recommended storage temperature), 5°C, and under stress conditions at 25°C/65% RH and 40°C/75% RH. Right panel: rgp41 stability monitored on three

rgp41 parameters at the indicated month (M) after storage at < -70°C (recommended storage temperature): Proportion of the rgp41 remaining under its natural trimeric state or trimers measured by SE-UPLC, purity of the rgp41 trimer determined by RP-UPLC (absence of aggregates), and proportion of rgp41 with intact C-terminal cysteine (intact full length required for lipidation) evaluated by ESI-TOF MS. Based on these data, frozen APIs are expected to remain stable for few years, continuous stability monitoring is still ongoing.

Supplementary Methods

Key component of the vaccine or formulation	New QC methods developed / validated during MACIVIVA	Number of QC methods used for GMP lot release
HA<br>(Seqirus, Australia)	None / all assays validated by supplier	9
3M-052<br>(3M, USA)	None / all assays validated by supplier	18
N-MCC-DPPE<br>(Corden Pharma LLC, Switzerland)	None / all assays validated or verified by supplier	24
Lipid DOPC<br>Lipid DOPE<br>(Merck & Cie Switzerland)	None / all assays validated or verified by supplier	25
P1 peptide (P1)<br>NH2-SQTQQEKNEQELLELDKWAS LWNWFDITNWLWYIKLSC-COOH<br>(Bachem AG, Switzerland)	2 HPLC methods for purity (%) and impurities (%) characterizations / all assays validated or verified by supplier	19
Recombinant gp41 (rgp41)<br>NH2- MQARQLLSGIVQQQNNLLRAIE-AQQHLLQLTVWGIKQLQARILAVERYL-KDQQLSGGRGGSSLEQIWNHTTWME-WDREINNYTSLIHSLIEESQNQQE KNEQELLELDLEHHHHC-COOH<br>(PX'Therapeutics, France)	About 14 methods (list below not exhaustive)<br>− Coomassie blue stained SDS-PAGE for identity, purity (%) and proportion of monomers, dimers and trimers.<br>− SE-UPLC for trimeric state and aggregates.<br>− RP-UPLC for identity and purity (%).<br>− MS-ESI for identity, MW determination, and determination of intact C-terminal cysteine (%).<br>/ all assays validated or verified by supplier	14
Liquid virosomes<br>(Mymetics, Switzerland)	4 HPLC methods for quantifying P1, rgp41, 3M-052, OEG (traces) content in the virosome<br>/ all assays validated or verified	14
Sublingual tablets<br>(Catalent, UK)	None / all assays validated or verified, but 3 HPLC methods were adapted for quantifying P1, rgp41 and 3M-052 content	10
Oral and nasal powders<br>(Upperton, UK)	3 adapted HPLC methods for identity and quantification of P1, rgp41, 3M-052 content.<br>Powder particle size & Moisture content<br>/ all assays validated or verified by supplier	9

Supplementary Table 1. Overview of the number of tests and/or analyses required for GMP lot release of the key vaccine excipients and APIs, together with the new solid vaccine dosages. Note that some excipients were not listed in this table due to the composition complexity, such as

different salts, fish gelatin, mannitol, trehalose, alginate, etc. Several analytical methods were already existing for QC but had to be retested in the context of the new galenic virosomal formulations that represent a different matrix than before. This table also excludes the new manufacturing methods developed for the three pilot lines. Considering the high number of methods used for GMP lot release of the different GMP pilot lines and the industrial know-how specific for each manufacturer, details could not be disclosed in this manuscript and protocols cannot be shared.

Test criteria	Liquid virosomes (for further processing)	Sublingual tablets	Nasal powder	Oral powder
Visual appearance	Whitish, opalescent solution	White, freeze-dried units. Units must be sufficiently robust to be removed from the packaging without breaking	White, spray-dried powder	White, spray-dried powder
Antigen and adjuvant content	By HPLC	By HPLC	By HPLC	By HPLC
Hemagglutinin content	By SRID	By qualitative assay	By qualitative assay	By qualitative assay
Virosome size	By DLS or NTA	By DLS or NTA	By DLS or NTA	By DLS or NTA
Unit Weights	Not applicable	50 mg dosing weight lead to about 8.3 mg tablet	Not applicable	Not applicable
		500 mg dosing weight lead to about 83 mg tablet		
Powder particle size	Not applicable	Not applicable	Sympatec particle sizer Volume Mean Diameter (VMD) > 9 mm diameter	Sympatec particle sizer Volume Mean Diameter (VMD) > 9 mm diameter
Residual moisture content	Not applicable	By Karl Fischer titration Not more than 8%	Loss on Drying measured by Dynamic Vapor Sorption (DVC)- Not more than 5%	Loss on Drying measured by Dynamic Vapor Sorption (DVC)- Not more than 5%
Disintegration or dissolution time	Not applicable	Not more than 60 seconds	Not more than 60 seconds	Not more than 60 seconds
Endotoxin content	By LAL assay	By LAL assay	By LAL assay	By LAL assay
Microbiological Purity	Total Aerobic Microbial Count: ≤ 100 CFU/mL	Total Aerobic Microbial Count: ≤ 100 CFU/g	Total Aerobic Microbial Count: ≤ 100 CFU/g	Total Aerobic Microbial Count: ≤ 100 CFU/g
	Total Combined Yeasts/mould count: ≤ 10 CFU/mL	Total Combined Yeasts/mould count: ≤ 10 CFU/g	Total Combined Yeasts/mould count: ≤ 10 CFU/g	Total Combined Yeasts/mould count: ≤ 10 CFU/g
Absence of Pathogens	*Pseudomonas aeruginosa:* Absent in 1 mL	*Pseudomonas aeruginosa:* Absent in 1 g	*Pseudomonas aeruginosa:* Absent in 1 g	*Pseudomonas aeruginosa:* Absent in 1 g
	Staphylococcus aureus: Absent in 1 mL	*Staphylococcus aureus:* Absent in 1g	*Staphylococcus aureus:* Absent in 1g	*Staphylococcus aureus:* Absent in 1g
	Echerichia coli : Absent in 1 mL	Not applicable	Not applicable	Not applicable

Supplementary Table 2. Selection of additional analytical methods performed on the different vaccine forms for quality controls. Endotoxin content, microbiological purity and absence of pathogens are standard requests from the European Pharmacopoeia, and performed according to validated methods. Quality controls on vaccine composition and physical attributes were

analyzed according to the industrial standards with measures performed at least twice for obtaining confirmation on data reproducibility.

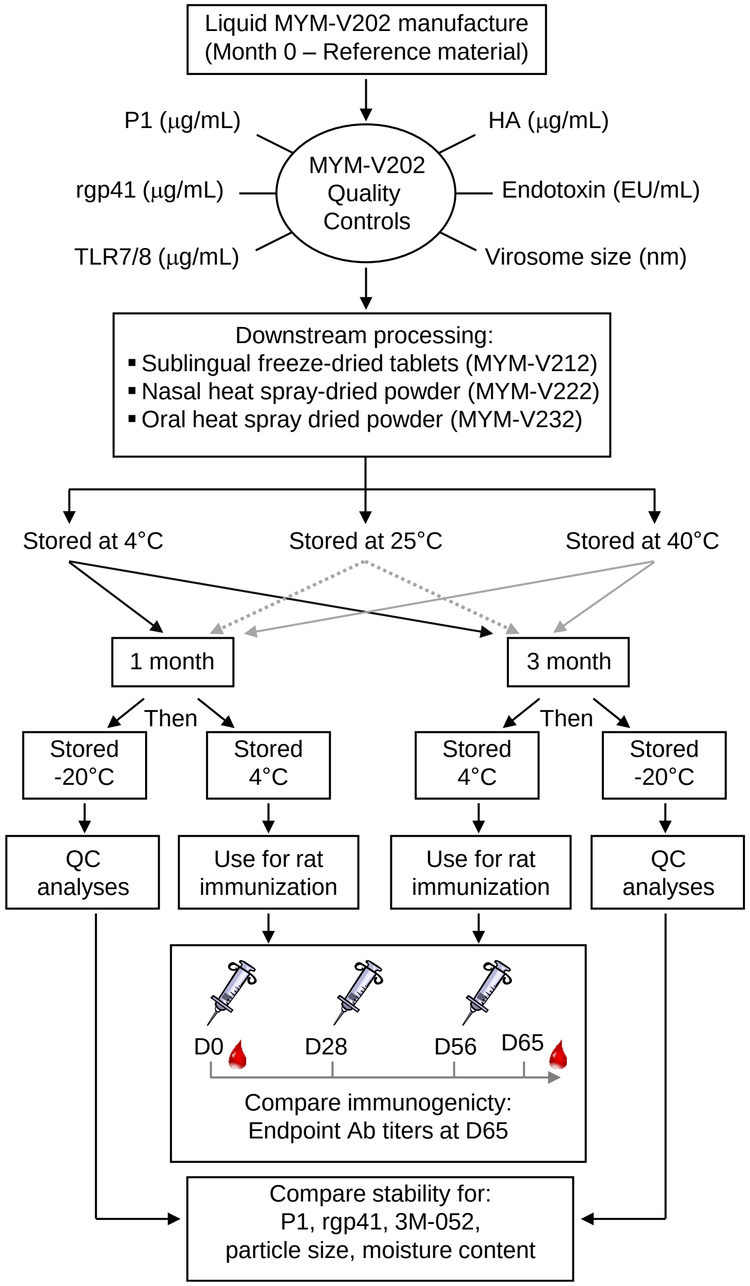

Supplementary Figure 2 Overview of the conducted analyses and investigations. The various vaccine forms were evaluate for the preservation of the initial vaccine properties and

immunogenicity during their storage under different environmental conditions. Results from these investigations are shown in the manuscript: Figure 5 for the virosome particle size, Figure 6 for the antigen content, and Figure 7 for the immunogenicity.

-

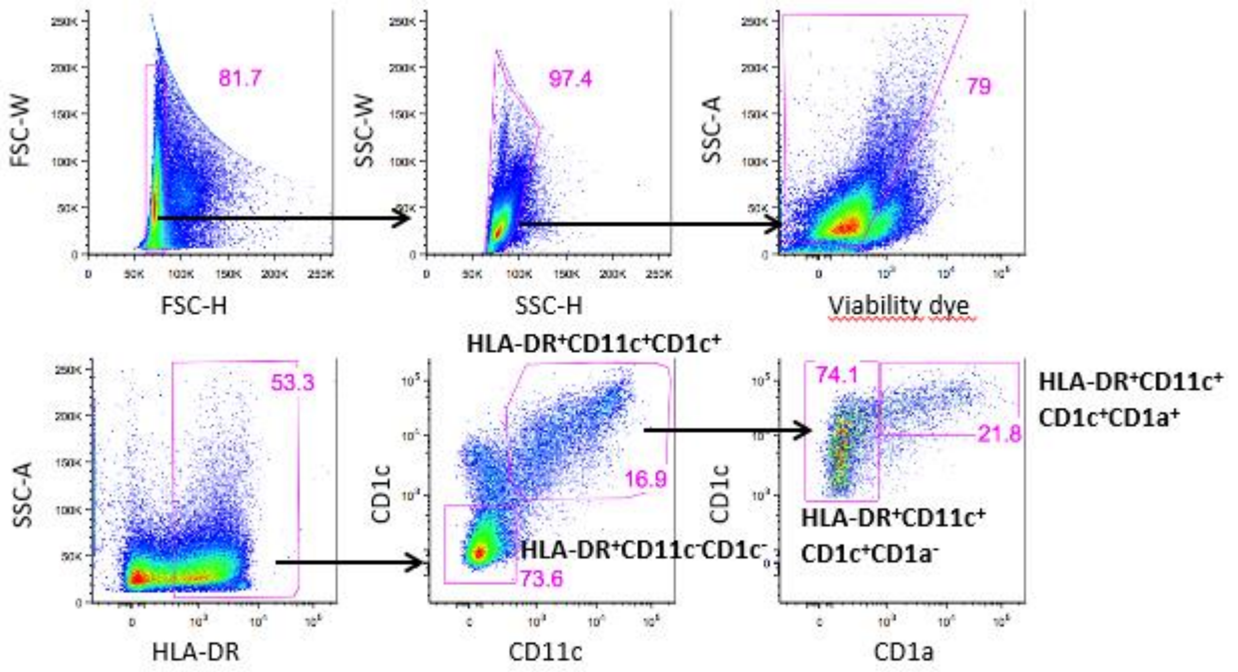

Supplementary Figure 3. Gating strategy for human CD34+ cells. Cells that are HLA-DR+CD11c- are not differentiated into dendritic cells and they represent the majority of the population and they are CD14$^-$. The subsequent gating is done cells HLA-DR+CD11c+CD1c+ to show the subpopulation CD1a+ and CD1a-. Cells that are HLA-DR+CD11c+ CD1c+CD1a+ have a phenotype similar to Langerhans cells, and cells that are HLA-DR+CD11c+CD1c+CD1a$^-$ are more similar to dermal dendritic cells. The fluorescent virosome-Atto 647 uptake and cell viability among the defined cell populations were determined.

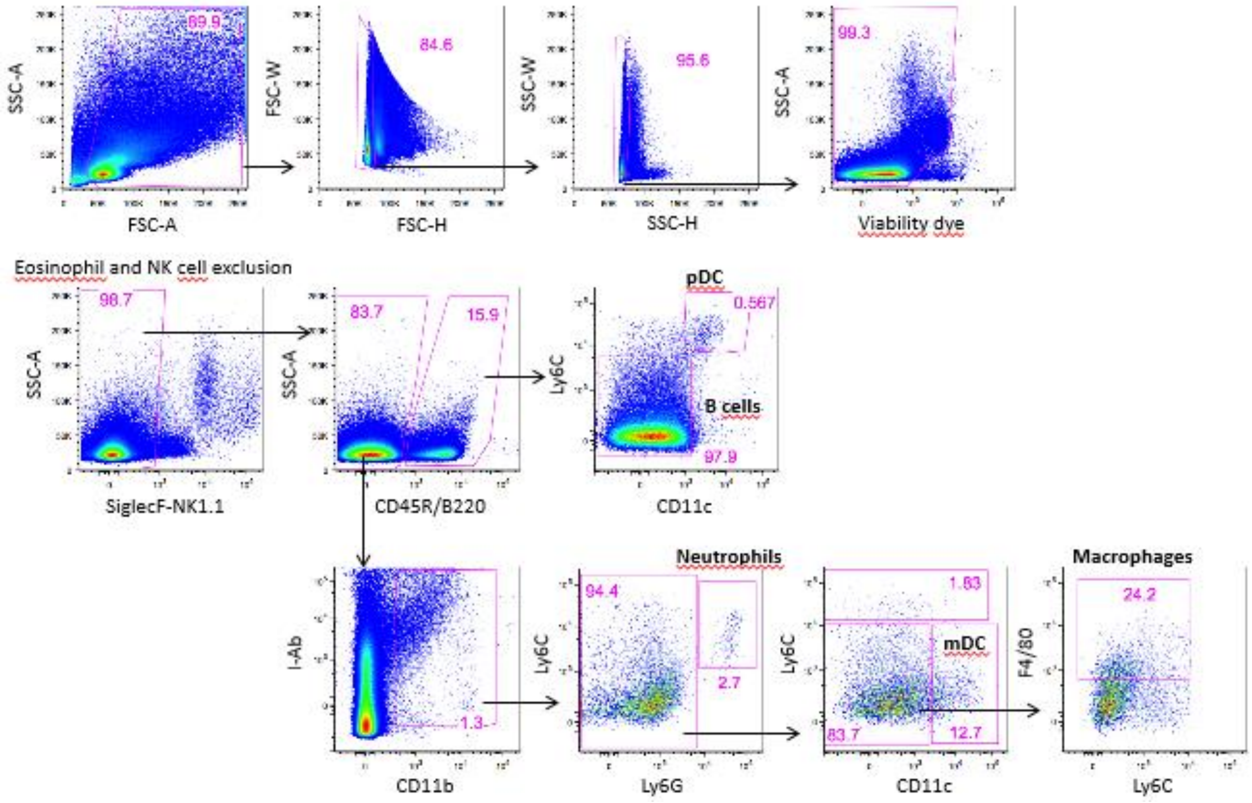

Supplementary Figure 4. Gating strategy for investigating the virosomes-Atto 647 migration to the mice draining lymph nodes. The gating was done first on the SSC/FSC parameters and then viable cells (LIVE/DEAD® viable dye) were selected for subsequent identification of the subpopulations defined by the following markers: B cells ($B220^{+}CD11C^{-}Ly6C^{-}$), Neutrophils ($CD11b^{+}Ly6C^{+}Ly6G^{+}$), Macrophages ($I\text{-}Ab^{+}CD11b^{+}F4/80^{+}Ly6C^{Lo}Ly6G^{-}CD11c^{-}$), Myeloid DC (mDC) ($I\text{-}Ab^{+}CD11b^{+}Ly6C^{Lo}Ly6G^{+}CD11c^{+}$), Plasmacytoid DC (pDC) ($I\text{-}Ab^{+}B220^{+}Ly6C^{+}CD11c^{+}$). Among each defined cell population, the absolute number of cells positive for virosomes-Atto 647 was determined.
